# Polyoxovanadate-alkoxide clusters as multi-electron charge carriers for symmetric non-aqueous redox flow batteries†
†Electronic supplementary information (ESI) available. See DOI: 10.1039/c7sc05295b


**DOI:** 10.1039/c7sc05295b

**Published:** 2018-01-08

**Authors:** L. E. VanGelder, A. M. Kosswattaarachchi, P. L. Forrestel, T. R. Cook, E. M. Matson

**Affiliations:** a Department of Chemistry , University of Rochester , Rochester , NY 14627 , USA . Email: matson@chem.rochester.edu; b Department of Chemistry , University at Buffalo , The State University of New York , Buffalo , NY 14260 , USA . Email: trcook@buffalo.edu

## Abstract

Non-aqueous redox flow batteries have emerged as promising systems for large-capacity, reversible energy storage, capable of meeting the variable demands of the electrical grid. Here, we investigate the potential for a series of Lindqvist polyoxovanadate-alkoxide (POV-alkoxide) clusters, [V_6_O_7_(OR)_12_] (R = CH_3_, C_2_H_5_), to serve as the electroactive species for a symmetric, non-aqueous redox flow battery. We demonstrate that the physical and electrochemical properties of these POV-alkoxides make them suitable for applications in redox flow batteries, as well as the ability for ligand modification at the bridging alkoxide moieties to yield significant improvements in cluster stability during charge–discharge cycling. Indeed, the metal–oxide core remains intact upon deep charge–discharge cycling, enabling extremely high coulombic efficiencies (∼97%) with minimal overpotential losses (∼0.3 V). Furthermore, the bulky POV-alkoxide demonstrates significant resistance to deleterious crossover, which will lead to improved lifetime and efficiency in a redox flow battery.

## Abstract 2

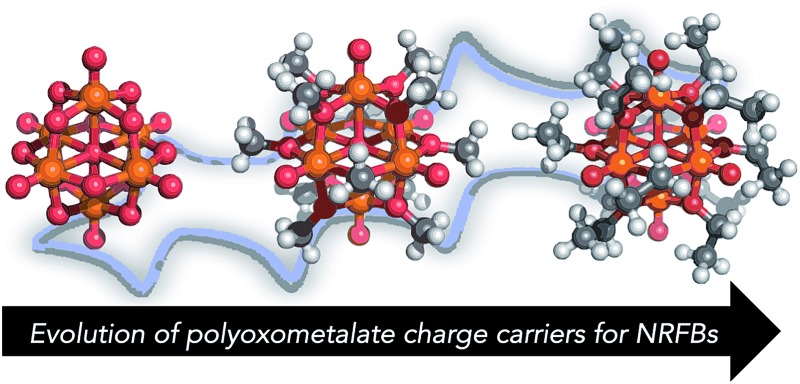
Installation of bridging alkoxide ligands leads to improvements to solubility, stability and redox profile of POM clusters!

## Introduction

Growing global energy demands drive the need for technologies that can address energy storage at the grid and microgrid scale, thereby enabling the incorporation of distributed renewable resources such as solar and wind.^1^ Redox flow batteries (RFBs) are an attractive approach, in that they decouple power and energy densities for straightforward scaling based on electrode stack size and reservoir volume. Unlike secondary batteries that contain solid-phase electrolytes and migratory ions as charge carriers, RFBs take advantage of electrolyte solutions typically consisting of a solvent, a supporting electrolyte, and an electroactive species that can cycle in its redox states.^1^–^3^ To date, viable devices have focused on aqueous electrolytes, which use inorganic salts as charge carriers.^2^ However, the narrow electrochemical window of water limits aqueous systems to a maximum attainable energy density of ∼130 kJ L^–1^.^4^

Non-aqueous redox flow batteries (NRFBs) can circumvent this limitation of aqueous systems, as solvent breakdown occurs only at extreme potentials.^5^,^6^ The use of non-aqueous media in a NRFB enables use of a wide library of active species, spanning molecules and materials, that are soluble in organic solutions.^5^ There are a myriad of small molecules that exhibit reversible redox chemistry, especially on short time scales. Computational methods^7^ and physical organic chemistry^8^ has been employed to narrow the scope of charge carriers to great effect; however, these studies have largely centred on redox active organic molecules—in part due to the computational accessibility of such structures.

A complementary physical inorganic strategy is equally powerful as a means to navigate the rich molecular space of redox active complexes and clusters. Coordination compounds have the added benefit over organic molecules of accessible d-orbitals on the metal centre, resulting in a series of charge states that range wide redox potentials.^9^–^11^ In such systems, it is possible to systematically tune physicochemical properties of relevance to NRFBs that include solubility, stability, and redox properties (*i.e.* reduction potentials and multi-electron transfer). These characteristics directly influence the energy density of a RFB, owing to the equation:1
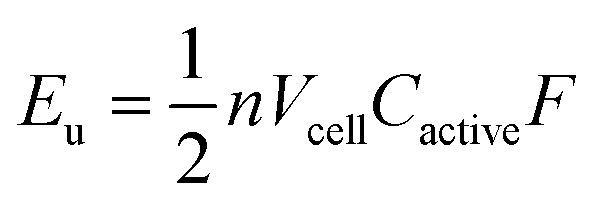
where *E*_u_ is volumetric energy density, *n* is the number of electrons transferred, *V*_cell_ is the average cell voltage, *C*_active_ is the concentration of the active species, and *F* is Faraday's constant.^3^ There are notable examples of using ligand design to enhance solubility, manifesting in higher *C*_active_ values. For example, a suite of M(acac)_3_ (M = V, Cr; acac = acetylacetonate-based ligands) complexes have been explored that span over four orders of magnitude in solubility.^12^ This strategy is not limited to a single coordination environment, as molecular solubilities have been similarly enhanced on metal–polypyridyl complexes using pendant ligand functionalization.^13^–^23^ Additional investigations into molecular modifications for improved energy densities of charge carriers include examples citing the use of redox active ligands in conjunction with metal ions for the generation of metal coordination compounds with large degrees of separation between redox waves.^13^–^25^ Most recently, the stability of metal coordination complexes has been addressed by the use of tridentate scaffolds.^8^ It is evident from the evolution of charge carriers in recent years that synthetic chemistry is crucial for the development of optimal electroactive materials for new NRFB technologies.

Exploiting the modular nature of transition metal complexes for RFBs is particularly effective when targeting multimetallic clusters such as polyoxometalates (POMs). These clusters consist of three or more transition metal oxyanions, linked together by bridging oxide units (O^2–^) to form three-dimensional structures, and are typically generated through high-yielding, self-assembly pathways.^26^ POMs have found many applications in the fields of catalysis and energy storage due to their unique physical and electrochemical properties.^27^–^31^ One exciting use of these polynuclear systems is their ability to serve as charge carriers for aqueous RFBs.^32^ By comparison, the area of non-aqueous redox flow battery development is underdeveloped for these systems. In seminal work, Anderson *et al.* made efforts to translate their aqueous POM charge-carrier, [SiV_3_W_9_O_40_]^7–^, to non-aqueous conditions, but found that the cluster exhibited minimal solubility and significant electrochemical instability in organic solvents.^33^ To exploit the advantageous properties of POMs, in combination with the enhanced potential windows of organic media, the identification of new metal–oxide clusters is required.

Recent studies from Barteau *et al.* highlight the multi-electron redox processes and broad potential windows of POMs, suggesting that with suitable molecular modifications, these polynuclear constructs could yield energy dense NRFB electrolytes.^34^,^35^ One such approach to the generation of metal–oxide clusters that meet the requirements of NRFB charge carriers is the integration of bridging alkoxide ligands (OR^–^) into the POM scaffold. This simple synthetic modification results in a retained homogeneity of the polynuclear clusters across all oxidation states, rendering POV-alkoxide clusters independent of counterions that typically govern the solubility of POMs.^36^,^37^ In addition to providing opportunities to modify the solubility of these systems (raising *C*_active_), the substitution of bridging alkoxide ligands can further tune the redox chemistry of the metal–oxide clusters, leading to increased electrochemical windows for these clusters (larger *V*_cell_).^32^,^38^ Additionally, although not included in eqn (1), the overarching gatekeeper for practical implementation as an active species in RFB technology is long-term stability across all charge-states.^39^,^40^ The ability to synthetically install ligands in a POM provides a direct route to affect chemical and redox stability. This molecular modification can be systematically controlled, thus providing opportunities to define structure–activity relationships that govern the redox behaviour of relevance to electrochemical energy storage.

**Fig. 1:**
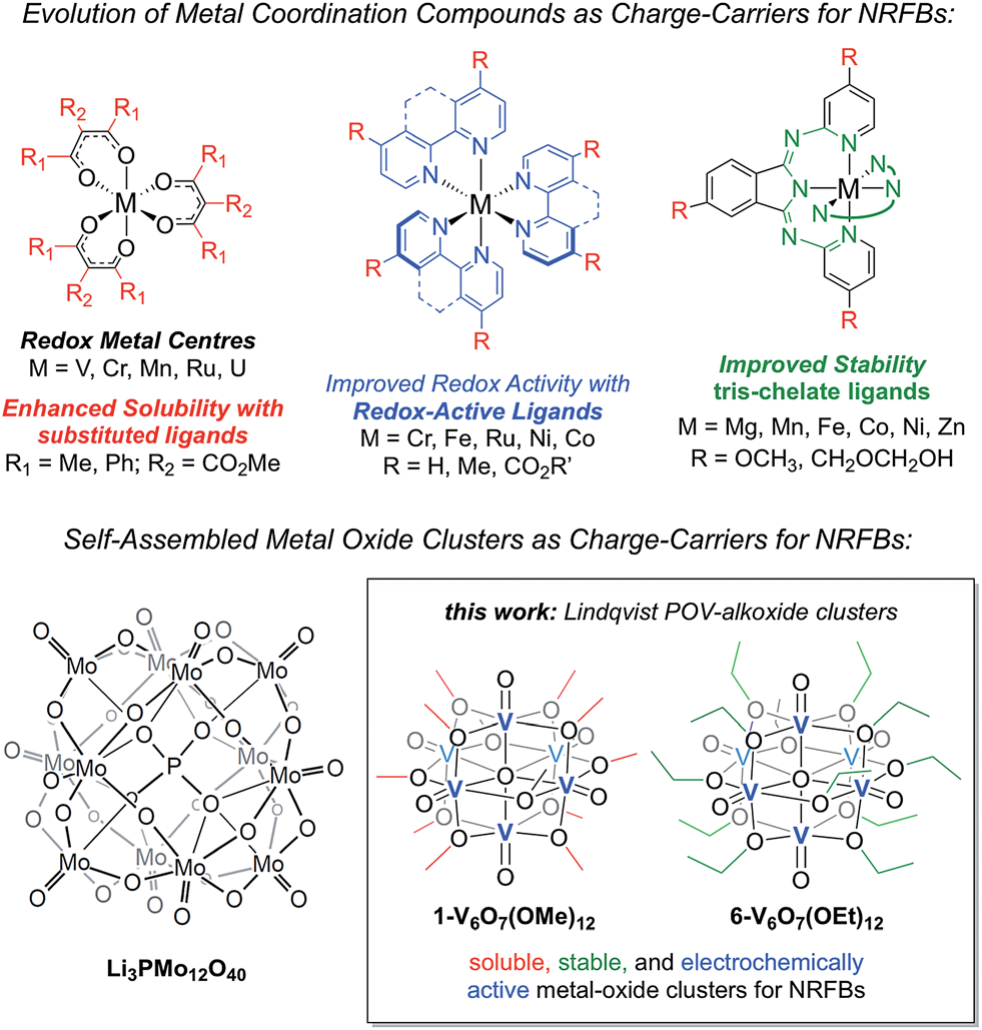
Evolution of metal coordination complexes and polyoxometalates as charge-carriers for non-aqueous redox flow batteries.

Herein, we present the use of mixed-valent, polyoxovanadate-alkoxide (POV-alkoxide) clusters, [V_6_O_7_(OR)_12_] (R = CH_3_, C_2_H_5_), whose bridging alkoxide ligands may be readily swapped from methoxide^41^ to ethoxide (Fig. 1).^42^,^43^ Both hexavanadate cores have been extensively characterized to evaluate their potential as NRFB charge carriers. Whereas the methoxide-functionalized polyoxovanadate cluster results in only modest energy storage potential, owing to its oxidative instability that limits the practical *V*_cell_, molecular modification to the ethoxide derivative provides access to the full multi-electron redox chemistry of the Lindqvist core. This work demonstrates a physical inorganic approach to the manipulation of molecular properties of POMs through the combination of synthetic chemistry and electrochemical analysis. The result is in a highly cyclable inorganic charge carrier for NRFBs, derived from earth-abundant elements and obtained from high-yielding, self-assembly methods.

## Results and discussion

### Physical and electronic properties of **1-V_6_O_7_(OCH_3_)_12_**

**Fig. 2:**
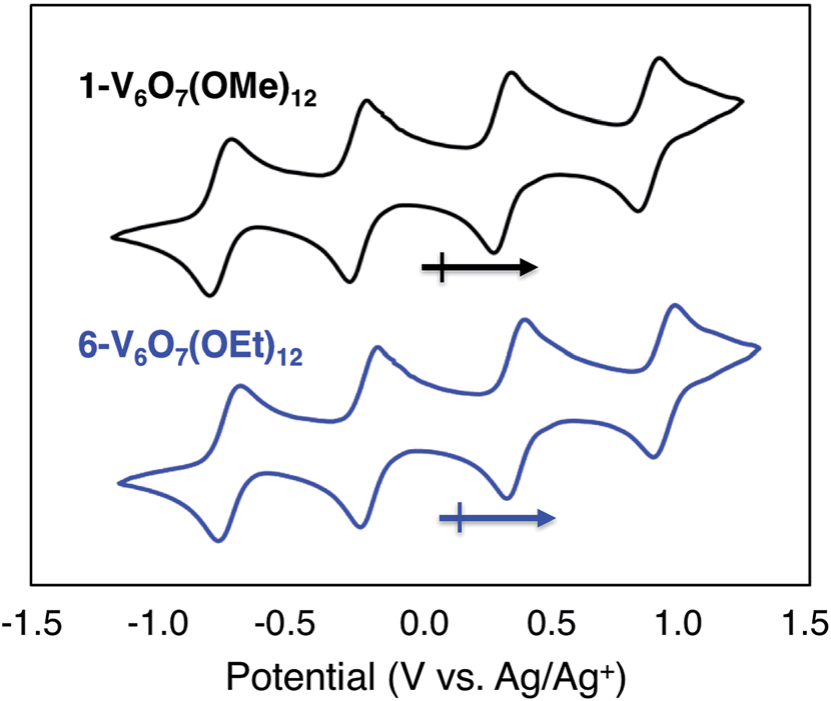
Cyclic voltammograms of 1 mM solutions of complexes **1-V_6_O_7_(OMe)_12_** (black) and **6-V_6_O_7_(OEt)**_12_ (blue) with 100 mM [NBu_4_][PF_6_] in MeCN and a scan rate of 200 mV s^–1^.

To serve as the electroactive species in an energy storage device, the molecules in various redox states must be highly soluble in organic media and simultaneously stable with respect to chemical decomposition, membrane crossover, and self-discharge. To investigate these properties for the family of POV-alkoxide clusters, we began by examining the electrochemical behaviour of [V^IV^_4_V^V^_2_O_7_(OCH_3_)_12_] (**1-V_6_O_7_(OMe)_12_**) in acetonitrile (MeCN). We observed similar redox behaviour for **1-V_6_O_7_(OMe)_12_** to that described by Hartl and coworkers, based on a cyclic voltammogram (CV) possessing four, one-electron, reversible redox events, with half-wave potentials (*E*_1/2_) ranging from –0.72 to +0.85 V *vs.* Ag/Ag^+^ (Fig. 2, Table 1). The ability for **1-V_6_O_7_(OMe)_12_** to undergo both oxidative and reductive processes enables its use in a symmetric RFB scheme, wherein a single molecule serves as both catholyte and anolyte.^44^,^45^ The wide separation between the outermost redox events (Δ*E* = 1.6 V), coupled with the ability of the cluster to hold four electrons, frames this POV-alkoxide as an effective charge carrier for flow batteries, provided that all redox states remain soluble and stable on times scales of relevance to electrochemical energy storage.

**Table 1:** Electrochemical parameters of complexes **1-V_6_O_7_(OMe)_12_** and **6-V_6_O_7_(OEt)_12_**

Complex	**1-V_6_O_7_(OCH_3_)_12_**			**6-V_6_O_7_(OEt)_12_**		
	*E*_1/2_ (V)	*D*_o_ (cm^2^ s^–1^)	*K*_o_ (cm s^–1^)	*E*_1/2_ (V)	*D*_o_ (cm^2^ s^–1^)	*K*_o_ (cm s^–1^)
[V^IV^_5_V^V^(OR)_12_]^1–^ + e^–^ ⇋ [V^IV^_6_O_7_(OR)_12_]^2–^	–0.72	4.88 × 10^–6^	8.16 × 10^–3^	–0.88	3.26 × 10^–6^	9.64 × 10^–2^
[V^IV^_4_V^V^_2_(OR)_12_]^0^ + e^–^ ⇋ [V^IV^_5_V^V^O_7_(OR)_12_]^1–^	–0.22	1.20 × 10^–5^	6.82 × 10^–2^	–0.34	1.45 × 10^–6^	1.01 × 10^–1^
[V^IV^_4_V^V^_2_O_7_(OR)_12_]^0^ ⇋ [V^IV^_3_V^V^_3_(OR)_12_]^1+^ + e^–^	+0.30	1.24 × 10^–5^	9.01 × 10^–2^	+0.22	1.45 × 10^–6^	4.50 × 10^–2^
[V^IV^_3_V^V^_3_O_7_(OR)_12_]^1+^ ⇋ [V^IV^_2_V^V^_4_(OR)_12_]^2+^ + e^–^	+0.85	9.05 × 10^–6^	1.01 × 10^–1^	+0.79	3.26 × 10^–6^	1.17 × 10^–1^

To obtain a preliminary measure of the electrochemical stability of this system, CV cycling of **1-V_6_O_7_(OMe)_12_** was conducted in MeCN. Following fifty cycles (∼1 hour), the CV of **1-V_6_O_7_(OMe)_12_** was largely unchanged, indicating good electrochemical stability on the time-scale of a CV experiment (Fig. S1†). To further investigate the redox behaviour of **1-V_6_O_7_(OMe)_12_**, CVs isolating each redox event were obtained at scan rates ranging from 100 to 2000 mV s^–1^ (Fig. S2†). A linear relationship between the peak currents of each event and the square root of the scan rate was observed, indicating a well-defined, mass-transfer limited process (Fig. S3–S6†).^46^,^47^ Thus, the Randles–Sevcik equation could be used to determine the diffusion coefficients for the redox series of POV-alkoxide clusters (Table 1).^48^–^50^ Next, the peak separation, Δ*E*_p_, was determined for each event, and plotted as a function of the square root of the scan rate (Fig. S3–S6†). The increase in Δ*E*_p_ at higher scan rates indicates that each of the four redox events is quasi-reversible.^51^ From these data, the Nicholson method was used to determine the heterogeneous electron transfer rate constants for each event (Table 1).^52^ These values are relatively fast when compared to previous redox flow battery electrolytes, indicating that galvanostatic charging will not be hindered by sluggish electron transfer.^53^,^54^

To establish the long-term, solution-phase stability of the various charge-states of complex **1-V_6_O_7_(OMe)_12_**, the chemical properties of the reduced and oxidized derivatives of the hexavanadate cluster were investigated. An active species must exhibit both electrochemical stability and chemical stability for electrolyte solution longevity. Electrochemical instability manifests in self-discharge processes, wherein the active species remains intact but undergoes redox reactions that reduce the stored charge. Chemical instability is more damaging in that the active species undergoes a change to its molecular structure, altering its redox behaviour. In an ideal redox flow cell featuring **1-V_6_O_7_(OMe)_12_** as the electroactive material, all five oxidation states of the molecule would be accessed throughout the multi-electron charge and discharge cycling. To evaluate the stability of the redox series, the reduced and oxidized derivatives of the hexavanadate cluster, [V^IV^_6_O_7_(OCH_3_)_12_]^2–^ (**2-V_6_O_7_(OMe)_12_^2–^**), [V^IV^_5_V^V^O_7_(OCH_3_)_12_]^1–^ (**3-V_6_O_7_(OMe)_12_^1–^**), and [V^IV^_3_V^V^_3_O_7_(OCH_3_)_12_]^1+^ (**4-V_6_O_7_(OMe)_12_^1+^**) were generated *via* synthetic procedures modelled after literature precedent^43^,^55^ (see ESI† for experimental details). The stabilities of complexes **2–4** in solution after one week were established using electronic absorption spectroscopy and CV (Fig. S7–S10†). Little change was noted in the spectra of complexes **2–4**, suggesting significant electrochemical and chemical stability for each oxidation state of the POV-alkoxide cluster in solution.

Although four of the five available charge states suggested by the CV of **1-V_6_O_7_(OMe)_12_** can be synthetically isolated, attempts to chemically generate the dicationic species, [V^IV^_2_V^V^_4_O_7_(OCH_3_)_12_]^2+^ (**5-V_5_O_6_(OMe)_12_^2+^**), were unsuccessful. Given the electrochemical reversibility of this redox event in the CV of **1-V_6_O_7_(OMe)_12_**, we hypothesized that complex **5-V_5_O_6_(OMe)_12_^2+^** could be accessed *via* electrosynthesis. Bulk oxidation of **1-V_6_O_7_(OMe)_12_** at 1.1 V yielded a solution with an open circuit potential of 1.0 V, suggesting successful formation of the desired dicationic charge state (Fig. S11†); however, the CV of the resulting solution showed changes in both the potentials and reversibility of all redox waves. Based upon these experiments, we can conclude that complex **1-V_6_O_7_(OCH_3_)_12_** is not stable under highly oxidizing conditions.

The solubilities of each of the synthetically available redox derivatives of **1-V_6_O_7_(OMe)_12_** were determined using electronic absorption spectroscopy in a 0.1 M MeCN solution of tetrabutylammonium hexafluorophosphate ([NBu_4_][PF_6_]) (Fig. S12–S15†). The solubilities for the isolable charged states of the POV-alkoxide cluster (∼100–200 mM) are on the same order of magnitude as previously reported non-aqueous charge carriers.^13^,^15^,^48^–^50^ The solubility of **1-V_6_O_7_(OMe)_12_** distinguishes this cluster from previously explored POM-based electroactive materials, which demonstrate little solubility or stability in organic media.^33^,^35^ We credit this enhanced solubility to the bridging alkoxide ligands in **1-V_6_O_7_(OMe)_12_**, which serve to stabilize the low charge-state of this cluster as well as to cooperatively interact with the polar organic solvent, MeCN. The collective physical and electrochemical properties of **1-V_6_O_7_(OMe)_12_** motivate continued electrochemical analyses of the polynuclear charge carrier.

### Extended cycling of 1-V_6_O_7_(OMe)_12_

**Fig. 3:**
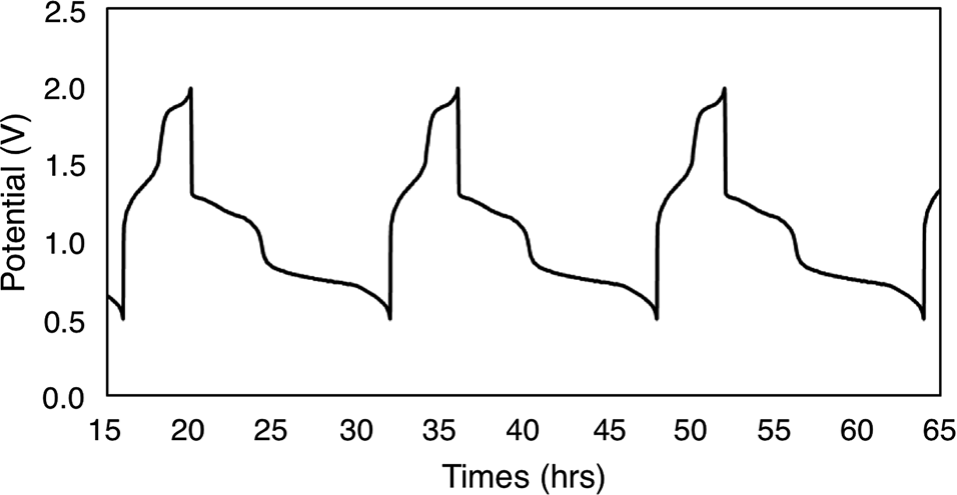
Potential curves of complex **1-V_6_O_7_(OMe)_12_** for cycles 2–4.

To evaluate the potential for **1-V_6_O_7_(OMe)_12_** to serve as the electroactive species in a NRFB, charge–discharge experiments were conducted in a static H-cell divided by an anion exchange membrane (AMI-7001) (Fig. 3). Prior to charging, each half of the H-cell contained 15 mL of a solution of 0.01 M **1-V_6_O_7_(OMe)_12_** with 0.5 M [NBu_4_][PF_6_] supporting electrolyte. Galvanostatic charging conditions were used, with a cut-off potential of 2.0 V, so as to include all four redox couples. A charging current of 0.2 mA and discharging current of 0.02 mA were selected based on preliminary experiments, with the lower discharge current intended to reduce overpotentials and ensure a complete discharge. Fig. 3 shows the trace of cycles 2–4 obtained from this experiment. The coulombic efficiency was ∼97% during cycling, with a state-of-charge for each cycle of ∼28%. Two plateaus are observed in both the charging and discharging traces, indicative of two separate electron transfer events. The charging plateaus at 1.4 V and 1.8 V fall to 0.8 V and 1.3 V, respectively, upon discharge of the cell, giving an average potential drop of ∼0.5 V. Potential losses during discharge in related systems have been attributed to mass-transport limitations and high internal resistance associated with cell design, which are similarly operative here.^56^,^57^

**Fig. 4:**
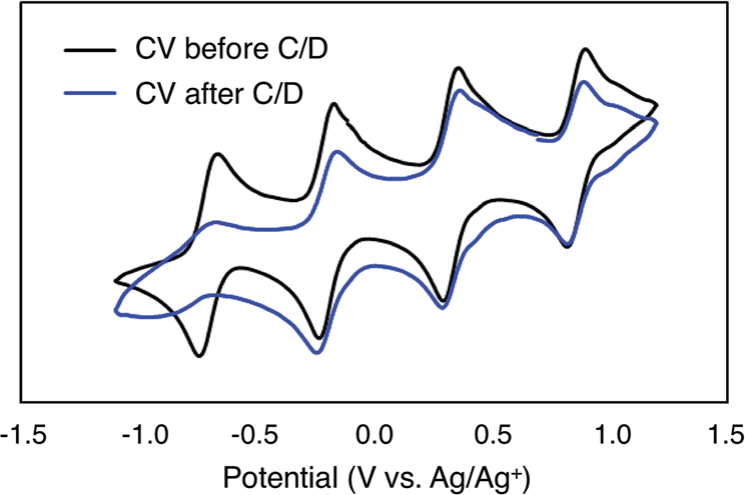
CV of complex **1-V_6_O_7_(OMe)_12_** before and after cycling.

To investigate the stability of the electroactive species, **1-V_6_O_7_(OMe)_12_**, during charge–discharge experiments, CVs of the anolyte and catholyte solutions were obtained following cycling. Although the catholyte solution showed no change based on its CV (Fig. S16†), the anolyte solution revealed potential shifts and loss of reversibility analogous to those observed following bulk oxidation of **1-V_6_O_7_(OMe)_12_** at +1.1 V (Fig. 4). This result is consistent with the previously discussed oxidative instability of complex **1-V_6_O_7_(OMe)_12_** under NRFB charging-schemes, limiting the effectiveness of the metal–oxide scaffold as an energy-dense charge carrier.

### Electrochemical optimization *via* ligand substitution

Given the oxidative instability of complex **1-V_6_O_7_(OMe)_12_** (*vide supra*) we hypothesized that the incorporation of bridging alkoxide ligands with a larger positive inductive effect would prevent active species degradation during cell cycling. The ethoxide-functionalized variant of the mixed-valent polyoxovanadate cluster, [V^IV^_4_V^V^_2_O_7_(OC_2_H_5_)_12_] (**6-V_6_O_7_(OEt)_12_**) has been reported,^42^,^43^ with redox potentials of the four quasi-reversible events shifted ∼–0.12 V from that of **1-V_6_O_7_(OMe)_12_** (Fig. 2, Table 1). The slight cathodic shift of the single electron redox events demonstrates the inductive effect that occurs as a consequence of ligand modification. The CV of complex **6-V_6_O_7_(OEt)_12_** shows good reversibility over 50 cycles (Fig. S17†), and diffusion coefficients, with heterogeneous electron transfer rate constants similar to those determined for **1-V_6_O_7_(OMe)_12_** (Table 1, Fig. S18–S21†). Collectively, these data suggest that this cluster exhibits good short-term electrochemical stability and kinetics, warranting further investigation of its function as a charge carrier for NRFBs.

As such, we set out to verify the physical and electrochemical properties of complex **6-V_6_O_7_(OEt)_12_**. The syntheses of the five redox states of the ethoxide-functionalized cluster, namely [V^IV^_6_O_7_(OC_2_H_5_)_12_]^2–^ (**7-V_6_O_7_(OEt)_12_^2–^**), [V^IV^_5_V^V^O_7_(OC_2_H_5_)_12_]^1–^ (**8-V_6_O_7_(OEt)_12_^1–^**), [V^IV^_3_V^V^_3_O_7_(OC_2_H_5_)_12_]^1+^ (**9-V_6_O_7_(OEt)_12_^1+^**), and [V^IV^_2_V^V^_4_O_6_(OC_2_H_5_)_12_]^2+^ (**10-V_6_O_7_(OEt)_12_^2+^**), were performed to verify their solubility and solution stability at longer time scales (Fig. S22–S26†). As the isolation of complexes **7-V_6_O_7_(OEt)_12_^2–^** and **8-V_6_O_7_(OEt)_12_^1–^** had not been reported previously, we independently synthesized these molecules *via* stoichiometric chemical reduction (see ESI† for experimental details). Characterization of the family of POV-alkoxide clusters (complexes **6–10**) *via* infrared and electronic absorption spectroscopies revealed the expected trends for sequential oxidation of the hexavanadate core, confirming isolation of each member of the redox series (Fig. S27†). Over the course of one week, complexes **6–10** showed no evidence of degradation based on CV and electronic absorption spectroscopy (Fig. S28–S32†). Results from these stability investigations suggest that, like its methoxide congener, the ethoxide-functionalized polyoxovanadate cluster has sufficient stability to serve as a charge carrier for NRFBs.

The most glaring deficiency in complex **1-V_6_O_7_(OMe)_12_** is the oxidative instability of the cluster core, rendering the system incapable of storing multiple electrons per charge carrier. In contrast to complex **1-V_6_O_7_(OMe)_12_**, Hartl and coworkers have presented the isolation of the mono- and di-cationic derivatives of the ethoxide-functionalized cluster, demonstrating the enhanced stability of this system under highly oxidizing conditions.^43^ To confirm the stability of the most-oxidized cluster under conditions relevant to cell cycling, bulk electrolysis was performed on a 0.01 M sample of **6-V_6_O_7_(OEt)_12_**. Following oxidation at +1.1 V, a CV of the resulting solution was identical to that of the neutral cluster, apart from its open circuit potential, which shifted to 1.0 V *vs.* Ag/Ag^+^ (Fig. S33†). This result suggests quantitative conversion to the desired oxidized species, **10-V_6_O_7_(OEt)_12_^2+^**, consistent with improvements to the stability of the hexavanadate, Lindqvist core following ligand substitution.

**Fig. 5:**
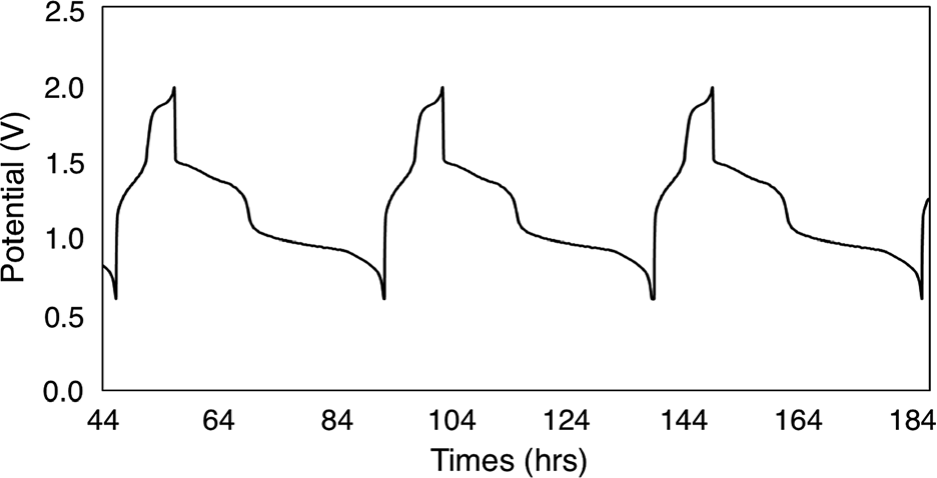
Potential curves of complex **6-V_6_O_7_(OEt)_12_** for cycles 2–4.

To continue our assessment of **6-V_6_O_7_(OEt)_12_**, we conducted charge–discharge cycling experiments using similar parameters to those selected for the previous cycling experiment with **1-V_6_O_7_(OMe)_12_**. Fig. 5 shows the trace for cycles 2–4 of this experiment, with plateaus that indicate a two-electron transfer occurs during both charging and discharging of the cell. The coulombic efficiency was ∼97% over the course of cycling, with a state-of-charge of ∼23%. A potential drop of ∼0.3 V was observed upon discharging the cell, indicating that overpotential losses were decreased by ∼0.2 V when **6-V_6_O_7_(OEt)_12_** was used in place of **1-V_6_O_7_(OMe)_12_**. This enhanced performance may be due to a better membrane compatibility for the larger cluster, resulting in a lower internal resistance, as discussed further below.

**Fig. 6:**
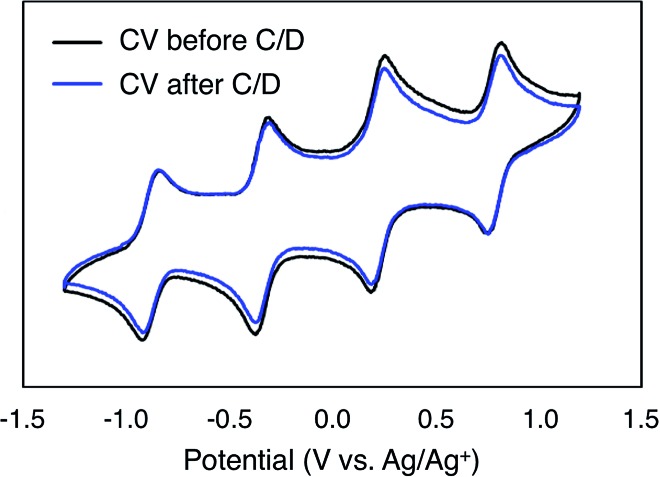
CV of complex **6-V_6_O_7_(OEt)_12_** before and after cycling.

To monitor the stability of **6-V_6_O_7_(OEt)_12_** during cycling, CVs of the electrolyte solutions were obtained before and after the experiment (Fig. 6). Following the charge–discharge experiment, we observe that the CV of both the catholyte and anolyte solutions remains unchanged. This result is in stark contrast with the CV of complex **1-V_6_O_7_(OMe)_12_** following charge–discharge experiments. This electrochemical data confirms that there is no decomposition of the active species throughout cycling for **6-V_6_O_7_(OEt)_12_**.

The charge–discharge cycles and electrochemical profiles of complexes **1-V_6_O_7_(OMe)_12_** and **6-V_6_O_7_(OEt)_12_** demonstrate the first example of multi-electron storage with metal–oxide clusters for NRFBs. Indeed, only one prior system has been reported for the application of POM clusters as charge-carriers in organic solvents. In recent work, Barteau and coworkers have summarized the successful implementation of a Keggin-type cluster, Li_3_[PMo_12_O_40_] as an electroactive material in MeCN (Fig. 1).^35^ Like the POV-alkoxide complexes reported here, the cluster exhibits remarkable stability and coulombic efficiency (97%) under charge-cycling conditions. However, due to the lack of bridging alkoxide ligands, Li_3_[PMo_12_O_40_] possesses modest solubility in acetonitrile (∼0.01 M), as compared to the POV-alkoxide clusters. Furthermore, the POM has a narrow potential window (*V*_cell_ = 0.36 V) and is capable of cycling only one electron, limiting the impact of this entry for non-aqueous flow battery charge carrier development. By comparison, the POV-alkoxide clusters possess *V*_cell_ values of ∼1.7 V, rivalling the electrochemical parameters of recently reported metal coordination complexes.^13^ The wide electrochemical window of this family of cluster compounds affords an opportunity to take advantage of the enhanced electrochemical stability of MeCN.

Furthermore, whereas ligand-based modifications have primarily been used to enhance charge carrier solubility,^12^,^14^ we have now demonstrated that simple substitutions of bridging alkoxide moieties (*i.e.* ethoxide for methoxide) can have a profound effect on the stability of a polynuclear, electroactive material. In the case of mononuclear metal coordination complexes, such ligand-based stabilizations require an entire redesign of the scaffold, often resulting in new coordination environments for the metal centre.^8^ In contrast, we have established that the stability of POV-alkoxide clusters may be controlled through facile alkoxide substitution, preserving the multi-electron redox activity of the hexavanadate core. This unique example of multi-electron storage across a readily modified metal–oxide scaffold clearly demonstrates the potential of alkoxide-bridged POMs to serve as superior charge carriers for emerging NRFB technologies.

### Crossover and membrane fouling

A difficulty facing all flow cell-based energy storage devices is the selection of membrane separators that are compatible with the electrolyte solutions.^58^,^59^ Two deleterious processes that can occur between charge carrier and membrane are membrane crossover and membrane fouling.^60^ In cells where the anolyte and catholyte solutions contain two different redox active species, membrane crossover results in cross-contamination that affects coulombic efficiency and leads to long-term fouling of the electrolyte solutions. This destructive property of asymmetric redox flow batteries can be obviated through the design of symmetric flow cells. In these systems, the same electroactive molecule functions at both the cathode and anode. That said, crossover within a symmetric cell still results in a decrease of the coulombic efficiency *via* deleterious charge-state recombination, even though the lifetimes of the electrolyte solutions are not affected.

In addition to the widely spaced redox events that allow POV-alkoxide clusters to serve as the active species for symmetric NRFB cells, these hexavanandate structures also benefit from an exceptionally large size relative to organic molecules and coordination complexes (∼9.3 Å and ∼12.0 Å in diameter for **1-V_6_O_7_(OMe)_12_** and **6-V_6_O_7_(OEt)_12_** respectively).^43^ We noted that during charge–discharge experiments with **1-V_6_O_7_(OMe)_12_**, cluster degradation was only observed in the anolyte solution, while the catholyte solution remained unaffected by charge cycling (Fig. 4 and S16†). Had there been significant crossover between these two half-cells, we would expect that the CV of both halves would be identical. This initial observation suggests the large size of the hexavanadate cluster prevents membrane crossover.

**Fig. 7:**
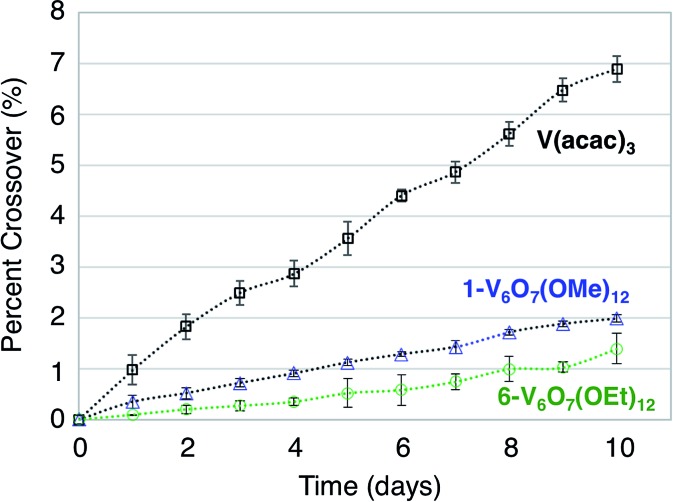
Extent of cross over for V(acac)_3_, **1-V_6_O_7_(OMe)_3_** and **6-V_6_O_7_(OEt)_3_**.

To further test this hypothesis, absorption spectroscopy experiments were performed to measure the extent of crossover of **1-V_6_O_7_(OMe)_12_** and **6-V_6_O_7_(OEt)_12_** through the H-cell membrane. Identical experiments were conducted using a mononuclear charge carrier, vanadium(iii) acetylacetonate (V(acac)_3_), which serves as a reference for comparison with the polynuclear metal–oxide clusters. V(acac)_3_ has established utility as a NRFB electrolyte, and is cited to partially mitigate crossover due to its bulky ligand framework (diameter of V(acac)_3_ = ∼9.8 Å).^50^,^61^ For each test, an H-cell, divided by an anion exchange membrane (AMI-7001), was assembled with one half-cell containing 0.05 M of the electroactive species (**1-V_6_O_7_(OMe)_12_**, **6-V_6_O_7_(OEt)_12_**, or V(acac)_3_) and 0.5 M [NBu_4_][PF_6_], while the other half-cell contained solely the electrolyte, 0.5 M [NBu_4_][PF_6_]. The solutions were stirred at ∼1000 rpm, and the concentration of each side of the solution was measured over 10 days using UV-vis spectroscopy. The average results over three trials are shown in Fig. 7. It is clear that not only does the bulky size of the POV-alkoxide clusters significantly reduce crossover when compared to V(acac)_3_, but the increase in size from the methoxide bridged **1-V_6_O_7_(OMe)_12_** to the ethoxide bridged **6-V_6_O_7_(OEt)_12_** further mitigates the extent of crossover observed in these systems. This result demonstrates that a straightforward, ligand substitution of bridging alkoxides on a self-assembled cluster can afford a measurable decrease in species crossover, thereby creating a NRFB cell with improved overall efficiency.

## Conclusions

In this report, we demonstrate how molecular control over POV-alkoxide clusters guides the formation of stable, multimetallic electroactive materials in non-aqueous media. The POV-alkoxide clusters reported are capable of cycling two electrons at both the anode and cathode of a symmetric H-cell. Although the methoxide-bridged cluster derivative **1-V_6_O_7_(OMe)_12_** shows oxidative instability, the substitution of bridging ethoxide ligands (**6-V_6_O_7_(OEt)_12_**) enhances the electrochemical properties of the hexavanandate core, resulting in a stable charge carrier. Furthermore, the large size of this series of POV-alkoxide clusters makes them resistant to membrane crossover, a feature that is also improved *via* ligand substitution from methoxide to ethoxide. Our combined synthetic and electroanalytic understanding of POV-alkoxides reveals the importance in cluster modifications, shedding light on the form–function relationships of this family of compounds with relevance to NRFBs. Current investigations in our laboratories are focused on further optimization of the physical and electrochemical properties of this class of polyoxometalates.

When evaluating the merit of new electroactive materials for NRFB applications, it is important to consider not only the physical properties of a complex, but also the accessibility of the molecule and feasibility of bulk synthesis. While previously reported metal coordination compounds have demonstrated fundamental advances in solubility as a result of systematic derivatization of the ligand, the assembly of these organic scaffolds requires multiple-step syntheses.^13^,^24^,^39^ In contrast, the POV-alkoxide clusters reported here are accessible through a one-step synthesis from earth-abundant, commercially available starting materials. The crystalline electroactive molecule can be isolated on gram-scales directly from the reaction mixture. This advantageous feature of these cluster complexes represents a departure in synthetic approaches to the generation of charge carriers for NRFB technologies.

## Conflicts of interest

There are no conflicts to declare.

## Supplementary Material

Supplementary information
