# Supplementary material for: Polyoxovanadate-alkoxide clusters as multi-electron charge carriers for symmetric non-aqueous redox flow batteries
Source: Chem Sci. 2018 Jan 8;9(6):1692–9. doi: 10.1039/c7sc05295b (PMC5890794; doi:10.1039/c7sc05295b)
Supplement: Supplementary file 1 [file SC-009-C7SC05295B-s001.pdf]

Electronic Supplementary Material (ESI) for Chemical Science.
This journal is © The Royal Society of Chemistry 2018

Electronic Supporting Information (ESI)

Polyoxovanadate-alkoxide Clusters as Multi-electron Charge Carriers for Symmetric Non-aqueous Redox Flow Batteries.

Lauren E. VanGelder[a], Anjula M. Kosswattaarachchi[b], Patrick L. Forrestel[a], Timothy R. Cook*[b], and Ellen M. Matson*[a]

[a]Department of Chemistry, University of Rochester, Rochester, New York 14627
[b]Department of Chemistry, University at Buffalo The State University of New York, Buffalo, New York 14260

Corresponding author emails: matson@chem.rochester.edu; trcook@buffalo.edu

Supporting Information Table of Contents:

General Considerations S3

POV-alkoxide Synthesis .... S3—S4

Electrochemistry .... S5—S6

Figure S1. Cyclic voltammetry cycling of **1-$V_6O_7(OMe)_{12}$** S7

Figure S2. CV of **1-$V_6O_7(OMe)_{12}$** at various scan rates S8

Figure S3-S6. Plots of i_p vs. $(v)^{1/2}$ and ΔE vs. $(v)^{1/2}$ for **1-$V_6O_7(OMe)_{12}$** .... S9—S12

Figure S7. Solution stability of **2-$V_6O_7(OMe)_{12}^{2-}$** S13

Figure S8. Solution stability of **3-$V_6O_7(OMe)_{12}^{-}$** S14

Figure S9. Solution stability of **1-$V_6O_7(OMe)_{12}$** S15

Figure S10. Solution stability of **4-$V_6O_7(OMe)_{12}^{+}$** S16

Figure S11. Bulk oxidation (+1.1 V) of **1-$V_6O_7(OMe)_{12}$** in MeCN S17

Figure S12. Solubility determination of **2-$V_6O_7(OMe)_{12}^{2-}$** S18

Figure S13. Solubility determination of **3-$V_6O_7(OMe)_{12}^{-}$** S19

Figure S14. Solubility determination of **1-$V_6O_7(OMe)_{12}$** S20

Figure S15. Solubility determination of **4-$V_6O_7(OMe)_{12}^{+}$** S21

Figure S16. CV of the catholyte **1-$V_6O_7(OMe)_{12}$** solution before and after CD cycling S22

Figure S17. Cyclic voltammetry cycling of **6-$V_6O_7(OEt)_{12}$** S22

Figure S18-S21. Plots of i_p vs. $(v)^{1/2}$ and ΔE vs. $(v)^{1/2}$ for **6-$V_6O_7(OEt)_{12}$** .... S23—S26

Figure S22. Solubility determination of **7-$V_6O_7(OEt)_{12}^{2-}$** S27

Figure S23. Solubility determination of **8-$V_6O_7(OEt)_{12}^{-}$** S28

Figure S24. Solubility determination of **6-$V_6O_7(OEt)_{12}$** S29

Figure S25. Solubility determination of **9-$V_6O_7(OEt)_{12}^{+}$** S30

Figure S26. Solubility determination of **10-$V_6O_7(OEt)_{12}^{2+}$** S31

Figure S27. UV-Vis and IR characterization of all redox isomers of **6-$V_6O_7(OEt)_{12}$** S32

Figure S28. Solution stability of **7-$V_6O_7(OEt)_{12}^{2-}$** S33

Figure S29. Solution stability of **8-$V_6O_7(OEt)_{12}^{-}$** S34

Figure S30. Solution stability of **6-$V_6O_7(OEt)_{12}$** S35

Figure S31. Solution stability of **9-$V_6O_7(OEt)_{12}^{+}$** S36

Figure S32. Solution stability of **10-$V_6O_7(OEt)_{12}^{2+}$** S37

Figure S33. Bulk oxidation (+1.1 V) of **1-$V_6O_7(OEt)_{12}$** in MeCN S38

References S39

Experimental Details

General considerations.

All manipulations were carried out in the absence of water and oxygen in a UniLab MBraun inert atmosphere glovebox under a dinitrogen atmosphere. Glassware was oven dried for a minimum of 4 hours and cooled in an evacuated antechamber prior to use in the drybox. Anhydrous methanol was purchased from Sigma-Aldrich and stored over activated 4 Å molecular sieves purchased from Fisher Scientific. All other solvents were dried and deoxygenated on a Glass Contour System (Pure Process Technology, LLC) and stored over activated 3 Å molecular sieves purchased from Fisher Scientific. $[NBu_4][BH_4]$ and $VO(O^iPr)_3$ were purchased from Sigma-Aldrich and used as received. $VO(OCH_3)_3$ was synthesized according to previous literature[1]. $[NBu_4][PF_6]$ was purchased from Sigma-Aldrich, recrystallized thrice using hot methanol, and stored under dynamic vacuum for a minimum of two days prior to use. ^1H NMR spectra were recorded at 500 on Bruker DPX-500 spectrometer locked on the signal of deuterated solvents. All chemical shifts were reported relative to the peak of residual ^1H signal in deuterated solvents. CD_3CN was purchased from Cambridge Isotope Laboratories, degassed by three freeze−pump−thaw cycles, and stored over activated 4 Å molecular sieves. Infrared (FT-IR, ATR) spectra of complexes were recorded on a Shimadzu IRAffinity-1 Fourier Transform Infrared Spectrophotometer and are reported in wavenumbers (cm^{-1}). Electronic absorption measurements were recorded at room temperature in anhydrous acetonitrile in a sealed 1 cm quartz cuvette with an Agilent Cary 60 UV-Vis spectrophotometer.

POV-alkoxide Syntheses.

The active species, **1-$V_6O_7(OMe)_{12}$** was accessed from one of two methods. The first is the previous synthetic route established by Hartl and coworkers[2], and the second is a modified procedure designed to access the molecule directly from commercially available reagents.

*Synthesis of **1-$V_6O_7(OMe)_{12}$** from commercial starting materials.*
In the glovebox, $VO(OCH(CH_3)_2)_3$ (510 mg, 2.08 mmol), $[NBu_4][BH_4]$ (53.7 mg, 0.208 mmol) and 14 mL methanol were charged in a 25 mL Teflon-lined Parr reactor. The Parr reactor was sealed, and the mixture heated in an over at 125 °C for 24 hours. The Parr reactor was allowed to cool to room temperature. Subsequent workup was conducted on the bench top. The resulting dark green solution was rotary evaporated to reduce the volume by half, then placed in a freezer at –35 °C for 24 hours. The resulting dark green crystals were spectroscopically pure **1-$V_6O_7(OMe)_{12}$** (231.289 mg, 0.293 mmol, 70.1%). The crystallization procedure was repeated twice to improve the yield (294.710 g, 0.373 mmol, 89.3 %). Characterization matches the previously reported synthesis.[2]

Complexes **2-$V_6O_7(OMe)_{12}^{2-}$** and **3-$V_6O_7(OMe)_{12}^{-}$** were prepared and characterized according to the literature.[3] The monocationic complex, **4-$V_6O_7(OMe)_{12}^{+}$**, has been reported previously[3], however was prepared here using an alternate method:

*Synthesis of **4-$V_6O_7(OMe)_{12}^{+}$**.*
In the glovebox, silver hexafluorophosphate ($AgPF_6$, 66 mg, 0.260 mmol) was added to a solution of **1-$V_6O_7(OMe)_{12}$** (200 mg, 0.253 mmol) in dichloromethane (10 mL). This solution was stirred for 1 hour, after which it was filtered and then evaporated to dryness. The green solid **4-$V_6O_7(OMe)_{12}^{+}$** was obtained in

good yield (184 mg, 0.196 mmol, 77.4 %). Characterization of this product matches that for the previously reported synthesis.[3]

Complex **6-$V_6O_7(OEt)_{12}$** was prepared and characterized according to the previous literature.[2]
Complexes **7-$V_6O_7(OEt)_{12}^{2-}$** and **8-$V_6O_7(OEt)_{12}^{-}$** had not been reported previously, and were prepared and characterized as follows:

Synthesis of ***7-$V_6O_7(OEt)_{12}^{2-}$****.*
In the glove box, $[NBu_4][BH_4]$ (108 mg, 0.420 mmol) was added to a solution of **6-$V_6O_7(OEt)_{12}$** (200 mg, 0.209 mmol) in acetonitrile (10 mL). This solution was allowed to stir for 1.5 hours, at which time the color had changed from dark green to teal blue. This solution was evaporated to half of its original volume and placed in the freezer. After 24 hours, dark blue-green crystals of **7-$V_6O_7(OEt)_{12}^{2-}$** precipitated from the solution (125 mg, 0.087 mmol, 41.6 %). ^{1}H NMR (400 MHz, CD_2Cl_2): δ = 1.00 (s) 1.49 (s) 1.64 (s), 3.37 (s) 26.34 (br). FT-IR (ATR, cm^{-1}) 737, 889, 922, 1063, 1170, 1183, 1474,1622,1673. UV-Vis (CH_3CN) [ε ($M^{-1}cm^{-1}$)]: 324 nm (5.150 x 10^3)

Synthesis of ***8-$V_6O_7(OEt)_{12}^{-}$****.*
In the glovebox, $VO(OC_2H_5)_3$ (392 mg, 1.939 mmol), $[NBu_4][BH_4]$ (105 mg, 0.408 mmol) and 14 mL methanol were charged in a 25 mL Teflon-lined Parr reactor. The Parr reactor was sealed, and the mixture heated in an over at 125 °C for 24 hours. The Parr reactor was allowed to cool to room temperature, and returned to the glove box. The resulting solution was evaporated to dryness, and washed with hexanes until the washes were colorless. The dark green solid **8-$V_6O_7(OEt)_{12}^{-}$** was obtained in good yield (295 mg, 0.246 mmol, 76.0%). ^{1}H NMR (400 MHz, CD_2Cl_2): δ = −1.59 (s) 1.01 (s) 1.64 (s) 3.43 (s) 23.42 (br). FT-IR (ATR, cm^{-1}) 734, 883, 951, 1028, 1169, 1379, 1460. UV-Vis (CH_3CN) [ε ($M^{-1}cm^{-1}$)]: 318 nm (5.877 x 10^3)

The syntheses of complexes **9-$V_6O_7(OEt)_{12}^{+}$** and **10-$V_6O_7(OEt)_{12}^{2+}$** were reported previously[2], however were prepared here using alternate synthetic routes:

Synthesis of ***9-$V_6O_7(OEt)_{12}^{+}$****.*
In the glovebox, silver hexafluorophosphate ($AgPF_6$, 53 mg, 0.210 mmol), was added to a solution of **6-$V_6O_7(OEt)_{12}$** (200 mg, 0.209 mmol) in dichloromethane (10 mL). The solution was allowed to stir for 1 hour, after which it was filtered and evaporated to dryness. This crude product was washed with hexanes (3x 10mL) leaving the green solid **9-$V_6O_7(OEt)_{12}^{+}$** (168 mg, 0.152 mmol, 72.7 %). Characterization of this product matches that for the previously reported synthesis.[2]

Synthesis of ***10-$V_6O_7(OEt)_{12}^{2+}$****.*
In the glovebox, nitrosonium hexafluorophosphate ($NOPF_6$, 74 mg, 0.420 mmol) was added to a solution of **6-$V_6O_7(OEt)_{12}$** (200 mg, 0.209 mmol) in dichloromethane (10 mL). The solution was allowed to stir for one hour, after which a solid green precipitate formed. This precipitate was collected via filtration and washed with ether (3x 10mL). The resulting light green solid **10-$V_6O_7(OEt)_{12}^{2+}$** was formed in moderate yield (59 mg, 0.047 mmol, 22.4 %). Characterization of this product matches that for the previously reported synthesis.[2]

Electrochemistry.

Cyclic voltammetry experiments.

Concentrations of POV-alkoxide and $[NBu_4][PF_6]$ used were 1 mM and 100 mM, respectively. CV measurements were carried out inside a nitrogen filled glove box (Vigor Tech, USA) using a Bio-Logic SP 200 potentiostat/galvanostat and the EC-Lab software suite. Cyclic voltammograms were recorded using a 3 mm diameter glassy carbon working electrode (CH Instruments, USA), a Pt wire auxiliary electrode (CH Instruments, USA), and a Ag/Ag^+ non-aqueous reference electrode with 0.01 M $AgNO_3$ in 0.05 M $[NBu_4][PF_6]$ in CH_3CN (BASi, USA). Cyclic voltammograms were *iR* compensated at 85% with impedance taken at 100 kHz using the ZIR tool included within the EC-Lab software.

Diffusion coefficients associated with each redox couple was determined by using the slope of the peak current (i_P) versus the square root of scan rate $\nu^{1/2}$. The Randles-Sevcik equation was used to estimate the diffusion coefficients from CV data. For a reversible redox couple, the peak current is given by the eq. S1;

$$i_p = 2.69 \times 10^5 n^{3/2} A\, c D_0^{1/2}\, \nu^{1/2} \qquad \text{Eq. S1}$$

In eq. S1, *n* is the number of electrons transferred; *A* is the electrode area (0.0707 cm^2 for the glassy carbon working electrode); *c* is the bulk concentration of the active species; D_0 is the diffusion coefficient of the active species; ν is the scan rate. For an irreversible redox couple, the peak current, is given by the eq. S2:

$$i_p = 2.99 \times 10^5 n^{3/2}\, \alpha^{1/2} A c D_0^{1/2}\, \nu^{1/2} \qquad \text{Eq. S2}$$

where α is the charge transfer coefficient (α ~ 0.5).

For the redox couples that show quasi-reversible kinetics, relationships for both reversible and irreversible redox reaction are usually employed to determine the diffusion coefficients of such redox processes. Therefore, an average value of diffusion coefficient was approximated for a quasi-reversible redox couple using both equations S1 and S2.[4]

The Heterogeneous Electron-Transfer (ET) Rate Constants were calculated using the Nicholson method.[5] The potential difference (*ΔEp*) of oxidation and reduction peaks were obtained at different scan rates. The transfer parameter, ψ, was extracted from the working curve constructed by Nicholson using *ΔEp* values. The standard heterogeneous charge transfers rate constant, k_0, for the electron transfer process was determined using the following equation:

$$\psi = \frac{k_0}{\left(\frac{\pi n F D \nu}{RT}\right)^{1/2}} \qquad \text{Eq. S3}$$

where n is the number of electrons transferred, *F* is the Faraday constant, *D* is the diffusion coefficient, *v* is the scan rate, *R* is the ideal gas constant and *T* is the temperature.[5-6]

Chronoamperometry/bulk electrolysis experiments

Bulk electrolysis experiments were performed in a H-cell with a glass frit separator (Porosity =10-16 µm, Adams and Chittenden, USA) using a Bio-Logic SP 200 potentiostat/galvanostat. An active species concentration of 1 mM was used. Working electrode compartment contained 5 mL of the active species with 100 mM $[NBu_4][PF_6]$ in CH_3CN and counter electrode compartment had 5 mL of 100 mM $[NBu_4][PF_6]$ in CH_3CN. A Pt mesh working electrode and a Pt wire counter electrode were used. Bulk electrolysis experiments were carried out using the chronoamperometry techniques available in EC lab software suite at constant potentials, selected from the CV data.

Charge-discharge experiments

Charge-discharge testing was done in a nitrogen filled glove box using a glass H-cell (Adams and Chittenden, USA) and a Bio-Logic SP 150 potentiostat/galvanostat. The electrolyte solutions used in charge-discharge experiments were 0.01 M active species in 0.5 M $[NBu_4][PF_6]$ in CH_3CN. Each compartment of the H-cell was filed with 15.0 mL of the electrolyte solution. The compartments of the H-cell were separated by an AMI-7001 anion exchange membrane (~0.05 cm^2, Membrane International Inc., USA). Two graphite felt electrodes (2 × 1 × 0.5 cm, Fuel Cell Store, USA) were placed in the posolyte and negolyte chambers. Electrodes attached to Pt wire current collectors submerged in the electrolyte solutions (~0.5 cm). Membranes and graphite felt electrodes were soaked in electrolyte solutions for 24 hours before each experiment. A galvanostatic charge-discharge method was adopted using a charging current of 0.2 mA and a discharging current of 0.02 mA for each experiment. The charge voltage cut offs were 2.0 V for the two separate experiments, both involving all four redox couples. The discharge cutoff voltage was 0.5 V for **1-$V_6O_7(OCH_3)_{12}$** and 0.6 V for **6-$V_6O_7(OC_2H_5)_{12}$**. For the duration of the charge-discharge experiments, both half cells were stirring at approximately 1,000 rpm.

Figure S1. CV cycling of 1.0 mM **1-$V_6O_7(OMe)_{12}$** in MeCN with 0.1 M $[NBu_4][PF_6]$ supporting electrolyte. Scan rate 100mV/s. Duration of cycling approximately one hour.

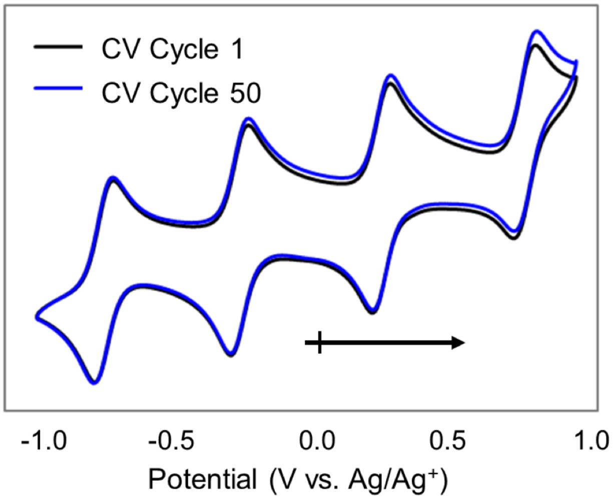

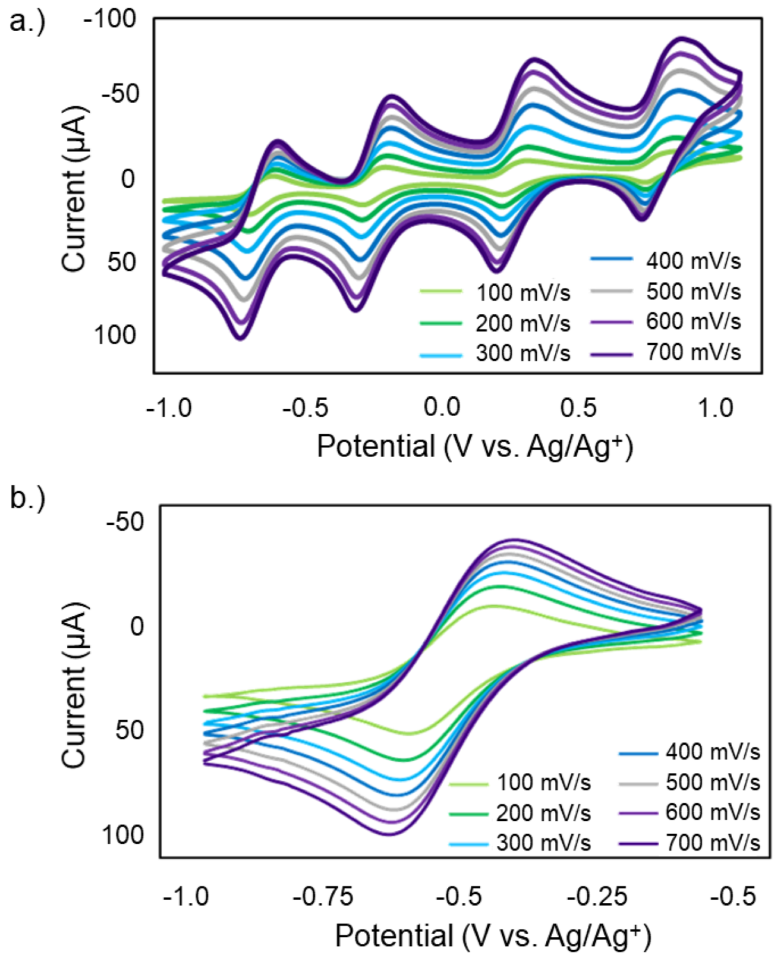

Figure S2. (a) CV of 1 mM **1-V_6O_7** in MeCN with 0.1 M $[NBu_4][PF_6]$ supporting electrolyte at various scan rates. (b) CV of only the most reducing redox-event (R2) of **1-V_6O_7**. Each redox event is isolated to determine i_p and ΔE values for use in diffusion coefficient (D_0) and heterogeneous electron transfer rate constant (k_0) calculations.

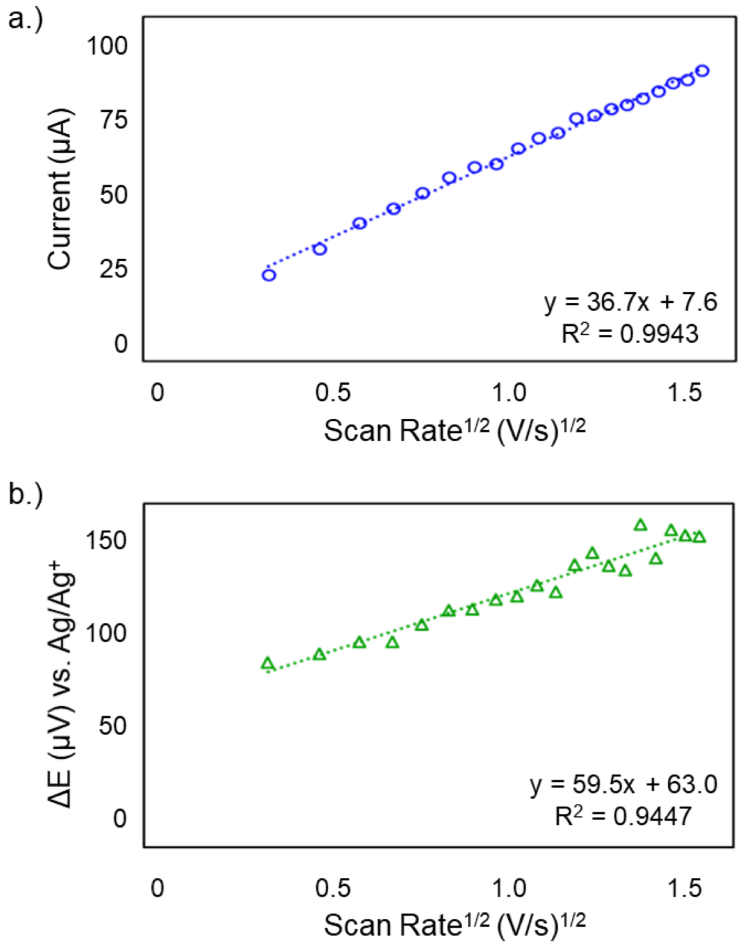

Figure S3. (a) Plots of i_p vs. $(v)^{1/2}$ and (b) ΔE vs. $(v)^{1/2}$ for the second reduction event ($E_{1/2}$ = -0.72V) of **1-$V_6O_7(OMe)_{12}$**

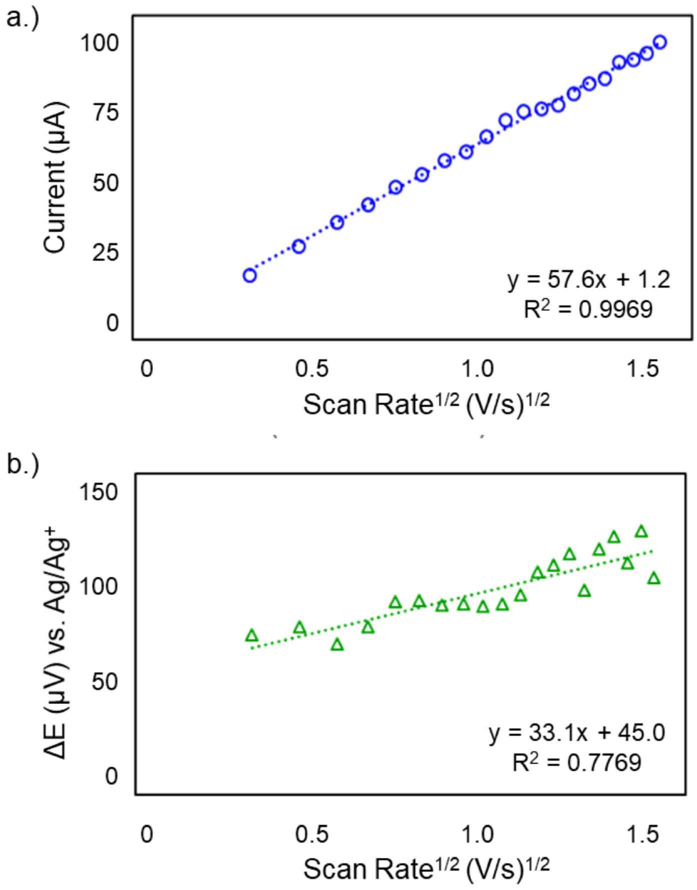

Figure S4. (a) Plots of i_p vs. $(v)^{1/2}$ and (b) ΔE vs. $(v)^{1/2}$ for the first reduction event ($E_{1/2}$ = -0.22 V) of **1-$V_6O_7(OMe)_{12}$**

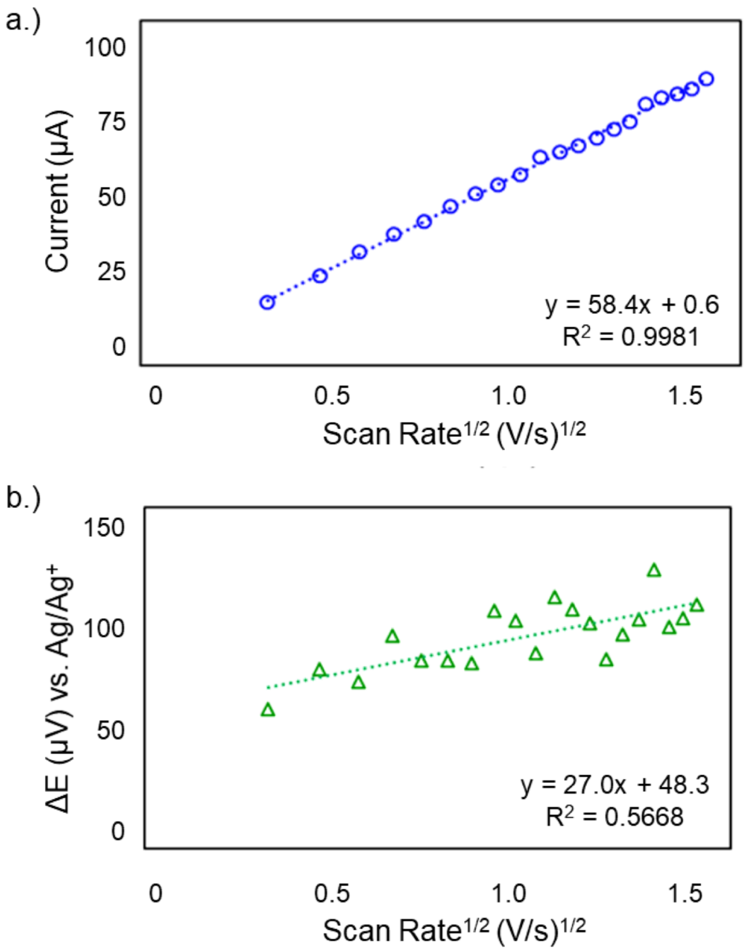

Figure S5. (a) Plots of i_p vs. $(v)^{1/2}$ and (b) ΔE vs. $(v)^{1/2}$ for the first oxidation event ($E_{1/2}$ = +0.30 V) of **1-$V_6O_7(OMe)_{12}$**

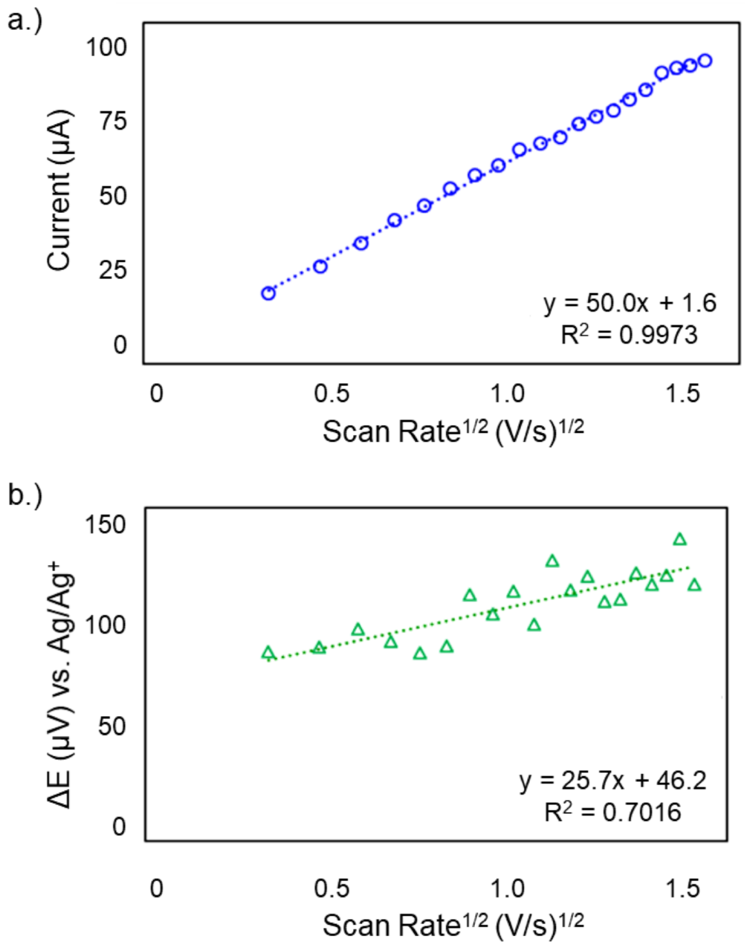

Figure S6. (a) Plots of i_p vs. $(v)^{1/2}$ and (b) ΔE vs. $(v)^{1/2}$ for second oxidation event ($E_{1/2}$ = +0.85 V) of **1-$V_6O_7(OMe)_{12}$**

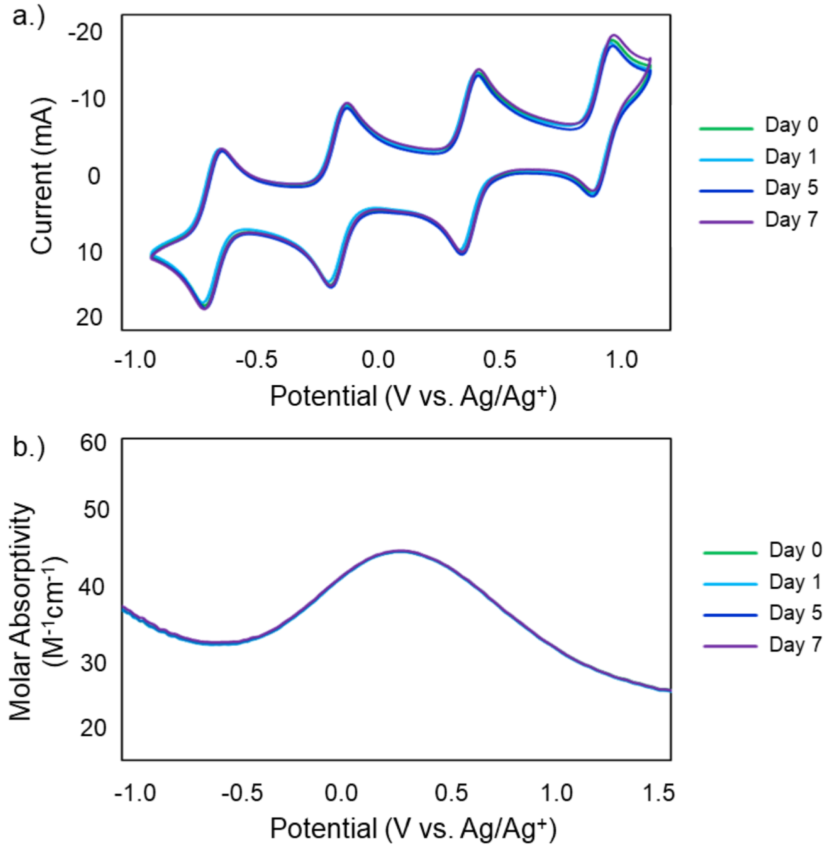

Figure S7. (a) Cyclic voltammograms at 100 mV/s and (b) absorption spectra of a 1 mM solution of **2-$V_6O_7(OMe)_{12}$**$^{2-}$ with 0.1 M $[NBu_4][PF_6]$ in acetonitrile over the course of a week.

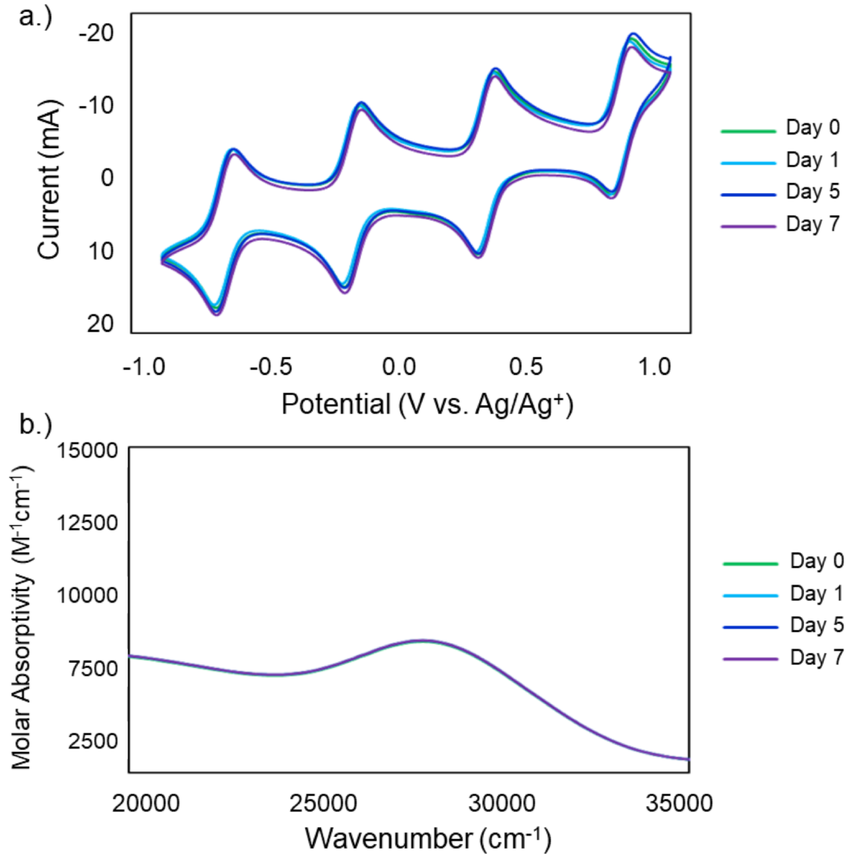

Figure S8. (a) Cyclic voltammograms at 100 mV/s and (b) absorption spectra of a 1 mM solution of **3-$V_6O_7(OMe)_{12}$**$^-$ with 0.1 M $[NBu_4][PF_6]$ in acetonitrile over the course of a week.

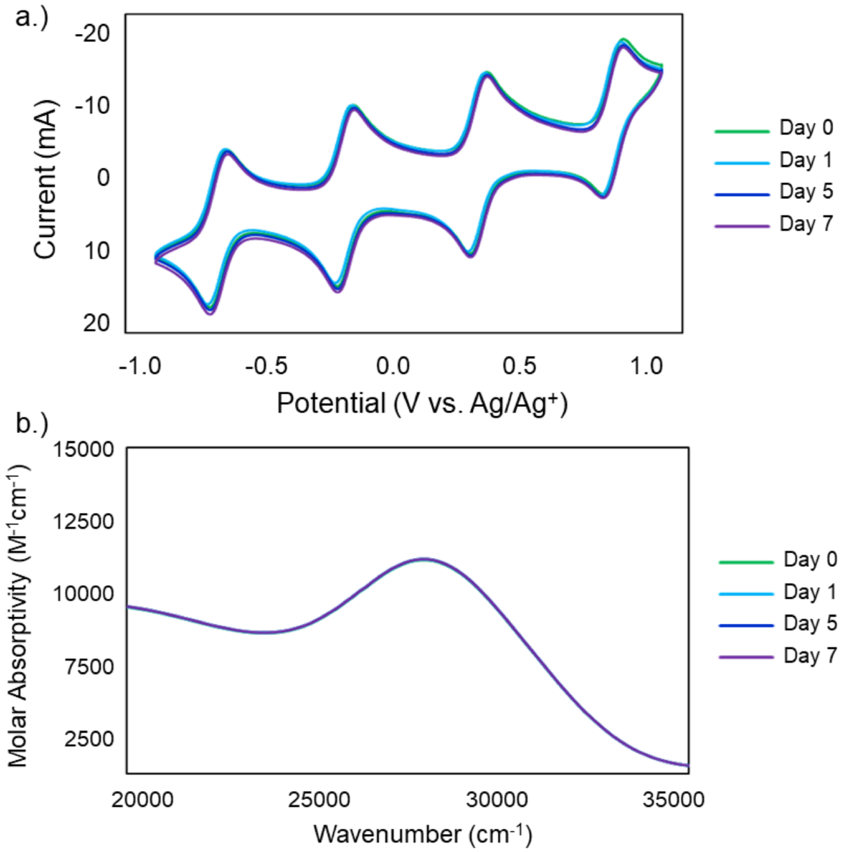

Figure S9. (a) Cyclic voltammograms at 100 mV/s and (b) absorption spectra of a 1 mM solution of **1-$V_6O_7(OMe)_{12}$** with 0.1 M $[NBu_4][PF_6]$ in acetonitrile over the course of a week.

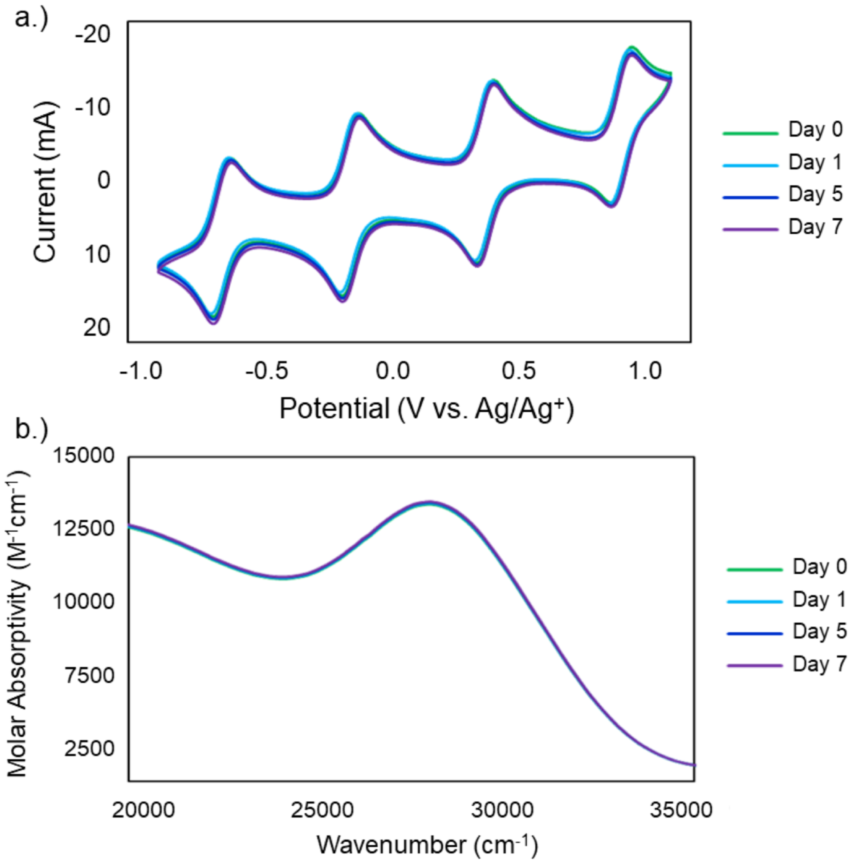

Figure S10. (a) Cyclic voltammograms at 100 mV/s and (b) absorption spectra of a 1 mM solution of **4-$V_6O_7(OMe)_{12}^+$** with 0.1 M $[NBu_4][PF_6]$ in acetonitrile over the course of a week.

Figure S11. Bulk oxidation (+1.1 V) of **1-$V_6O_7(OMe)_{12}$** in MeCN

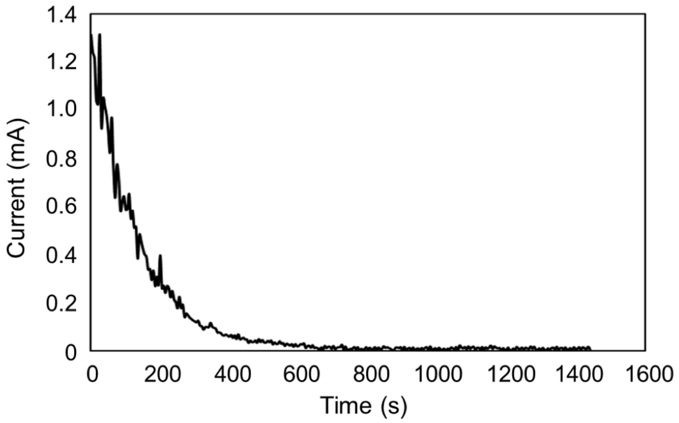

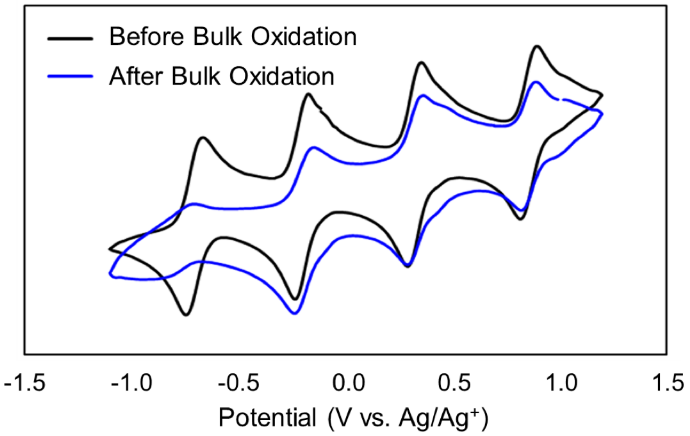

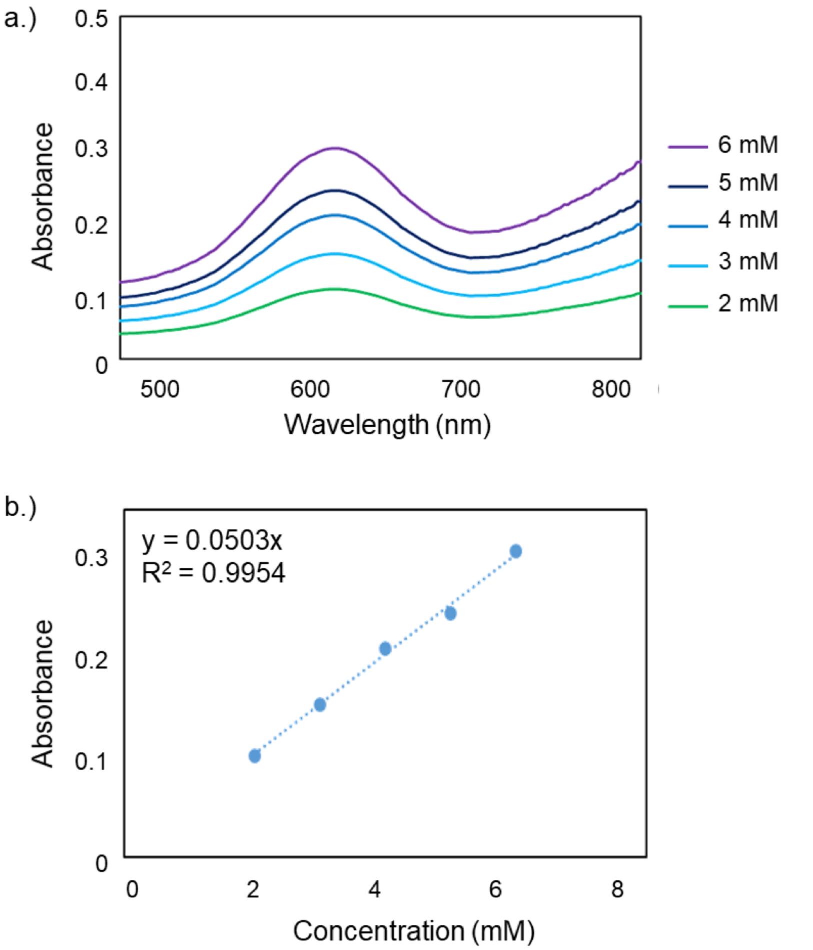

Figure S12. (a) absorbance spectra of **2-$V_6O_7(OMe)_{12}^{2-}$** at various concentrations in MeCN with 0.1 M $[NBu_4][PF_6]$ (b) Beer's Law plot of absorbance at 626 nm for **2-$V_6O_7(OMe)_{12}^{2-}$** in MeCN.

Molar Absorptivity at 626nm : 50.3 $M^{-1}cm^{-1}$

saturated solution 1:
Diluted 200uL to 5mL MeCN
A = 0.222605
C = 4.2001 E-3 M

5mL(M) = .200 mL X M

X = 0.105 M

saturated solution 2:
Diluted 200uL to 5mL MeCN
A = 0.22048
C = 4.16 E-3 M

5mL(M) = .200 mL X M

X = 0.104 M

saturated solution 3:
Diluted 200uL to 5mL MeCN
A = 0.20776
C = 3.9218 E-3 M

5mL(3.9218 E-3 M) = .200 mL X M

X = 0.098 M

Average solubility = 102 mM

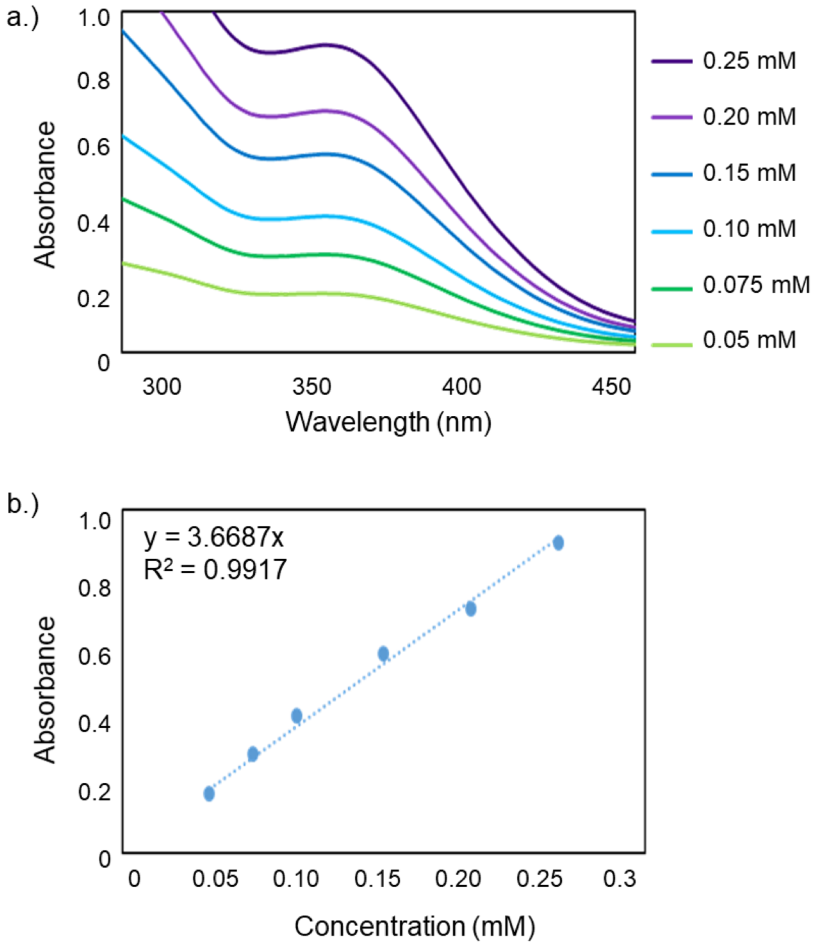

Figure S13. (a) absorbance spectra of **3-$V_6O_7(OMe)_{12}^-$** at various concentrations in MeCN with 0.1 M $[NBu_4][PF_6]$ (b) Beer's Law plot of absorbance at 378 nm for **3-$V_6O_7(OMe)_{12}^-$** in MeCN.

Molar Absorptivity at 378nm : 3,668.7 $M^{-1}cm^{-1}$

saturated solution 1:
Diluted 5uL to 5mL MeCN
A = .684129
C = 1.865 E-4

5mL(1.865 E-4 M) = .005 mL X M

X = 0.1865 M

saturated solution 2:
Diluted 5uL to 5mL MeCN
A = .693158
C = 1.889 E-4

5mL(1.889 E-4 M) = .005 mL X M

X = 0.1889 M

saturated solution 3:
Diluted 5uL to 5mL MeCN
A = .715259
C = 1.950 E-4

5mL(1.950 E-4 M) = .005 mL X M

X = 0.1950 M

Average solubility = 190 mM

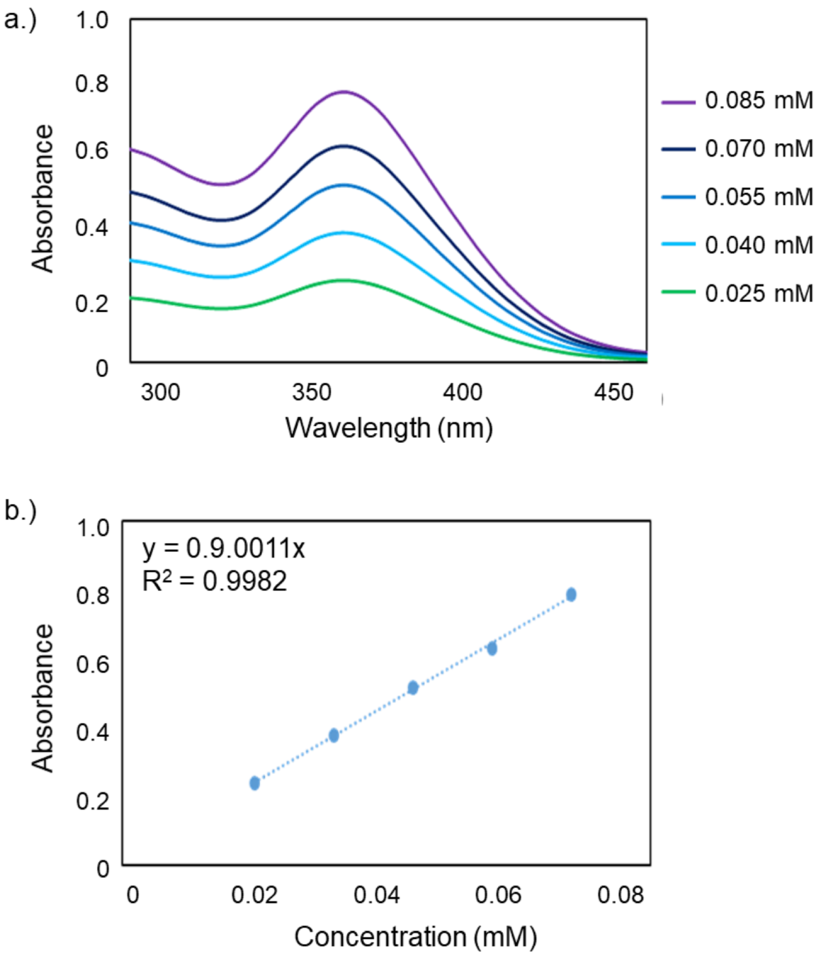

Figure S14. (a) absorbance spectra of **1-$V_6O_7(OMe)_{12}$** at various concentrations in MeCN with 0.1 M $[NBu_4][PF_6]$ (b) Beer's Law plot of absorbance at 382 for **1-$V_6O_7(OMe)_{12}$** in MeCN.

Molar Absorptivity at 382 nm : 9001.1 M^{-1} cm^{-1}

saturated solution 1:
Diluted 5uL to 10mL MeCN
A = 0.918122
C = 1.02 E-4 M

10mL(1.02 E-4 M) = .005 mL X M

X = .204 M

saturated solution 2:
Diluted 5uL to 10mL MeCN
A = 0.927113
C = 1.03 E-4

10mL(1.03 E-4 M) = .005 mL X M

X = .206 M

saturated solution 3:
Diluted 5uL to 10mL MeCN
A = 0.882108
C = 1.96 E-5 M

10mL(1.96 E-4 M) = .005 mL X M

X = .196 M

Average solubility = 202 mM

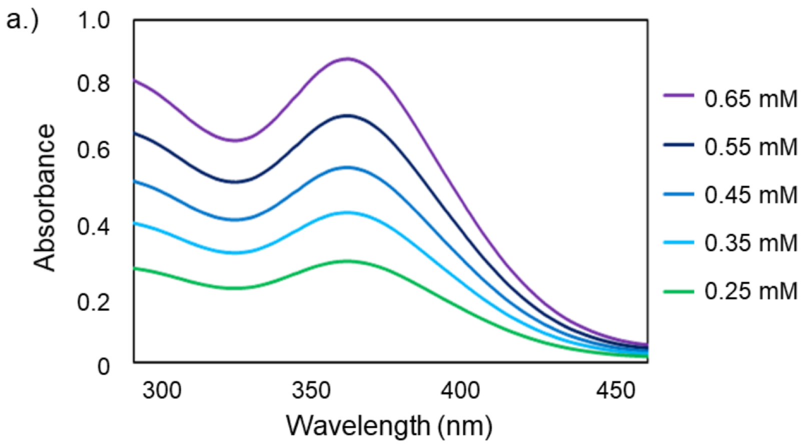

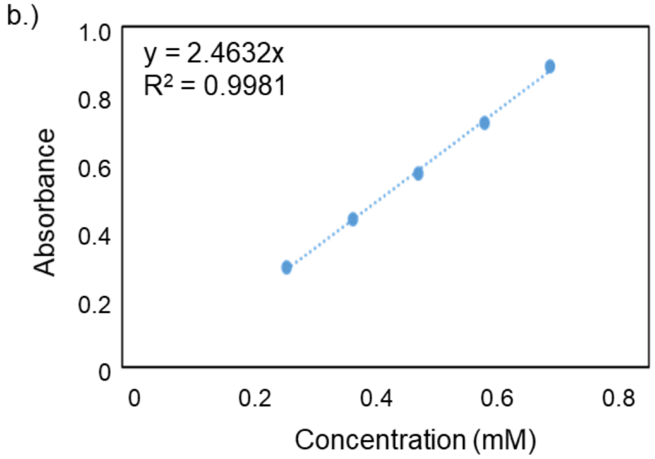

Figure S15. (a) absorbance spectra of **4-$V_6O_7(OMe)_{12}^+$** at various concentrations in MeCN with 0.1 M $[NBu_4][PF_6]$ (b) Beer's Law plot of absorbance at 382 for **4-$V_6O_7(OMe)_{12}^+$** in MeCN.

Molar Absorptivity at 382 nm : 14,632 $M^{-1}cm^{-1}$

saturated solution 1:
Diluted 2.5uL to 10mL MeCN
A = 0.68404
C = 4.675 E-5

10mL(4.675 E-5 M) = .0025 mL X M

X = 0.187 M

saturated solution 2:
Diluted 2.5uL to 10mL MeCN
A = 0.691362
C = 4.725 E-5 M

10mL(4.725 E-5 M) = .0025 mL X M

X = 0.189 M

saturated solution 3:
Diluted 2.5uL to 10mL MeCN
A = 0.643808
C = 4.4 E-5 M

10mL(4.4 E-5 M) = .0025 mL X M

X = 0.176 M

Average solubility = 184 mM

Figure S16. CV of catholyte solution of **1-$V_6O_7(OCH_3)_{12}$** before and after charge discharge cycling at 2 V cutoff potential.

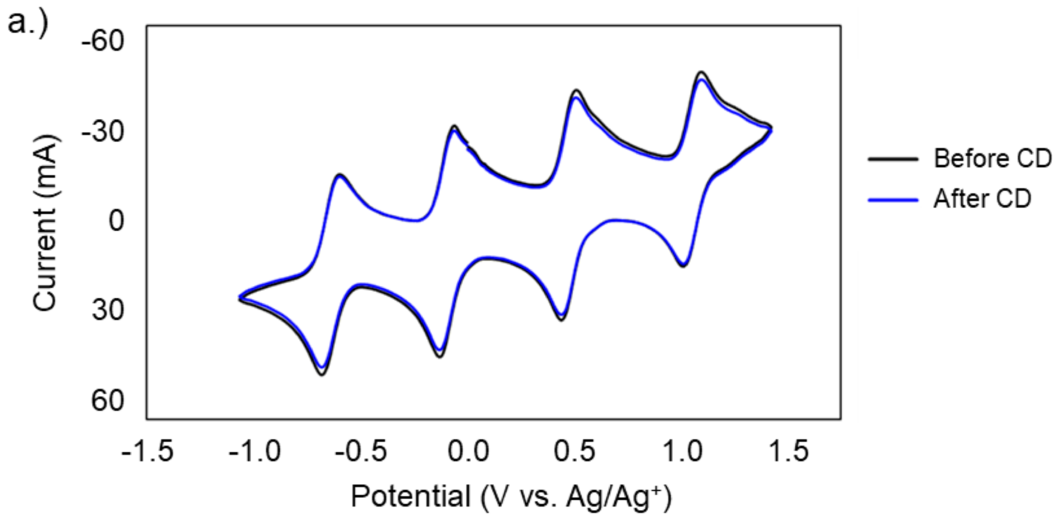

Figure S17. CV cycling of 1 mM **6-$V_6O_7(OEt)_{12}$** in acetonitrile with 0.1 M $[NBu_4][PF_6]$ supporting electrolyte

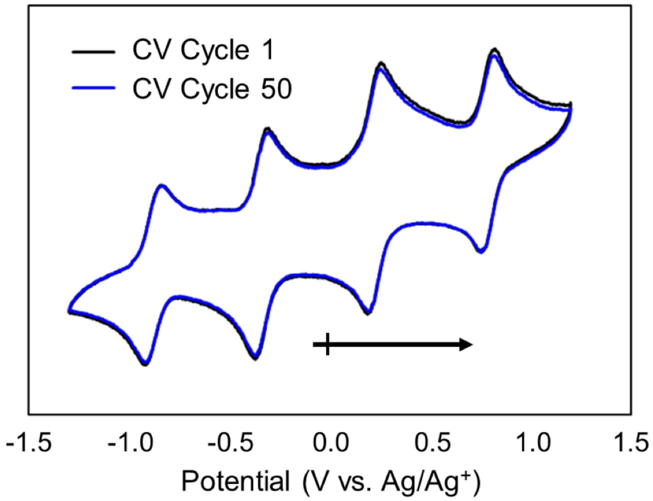

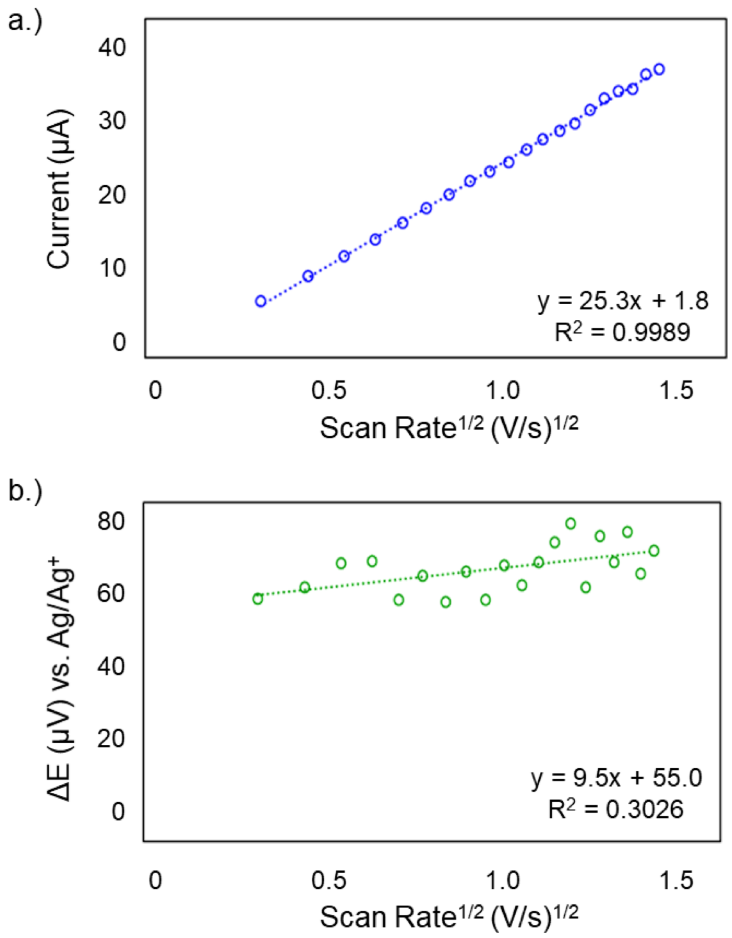

Figure S18. (a) Plots of i_p vs. $(v)^{1/2}$ and (b) ΔE vs. $(v)^{1/2}$ for the second reduction event ($E_{1/2}$ = -0.88 V) of **6-$V_6O_7(OEt)_{12}$**

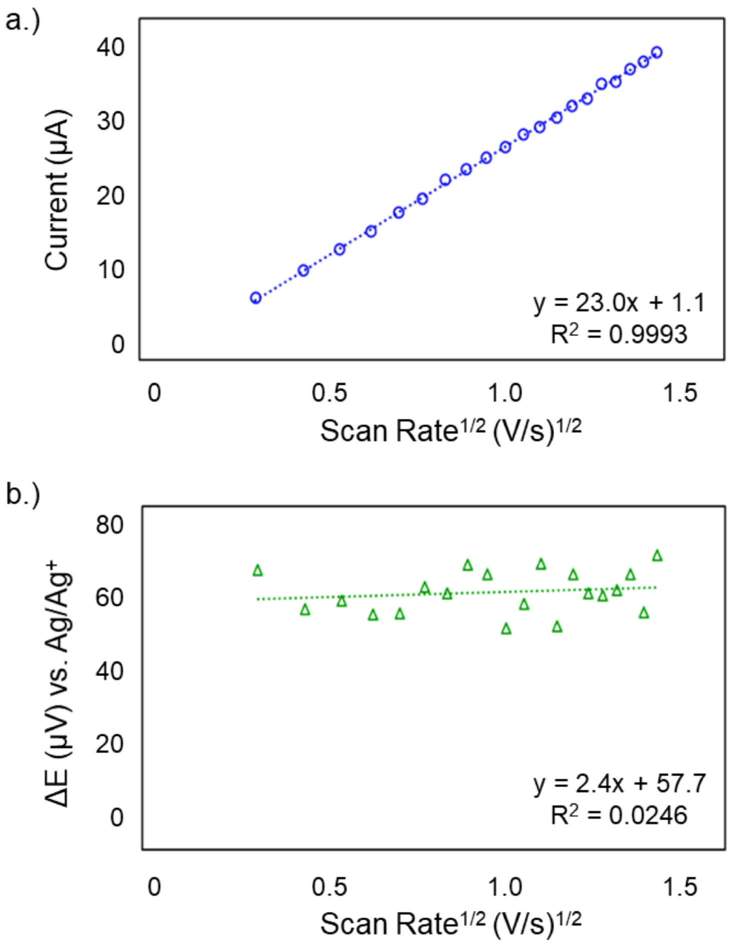

Figure S19. (a) Plots of i_p vs. $(v)^{1/2}$ and (b) ΔE vs. $(v)^{1/2}$ for the first reduction event ($E_{1/2}$ = -0.34 V) of **6-$V_6O_7(OEt)_{12}$**

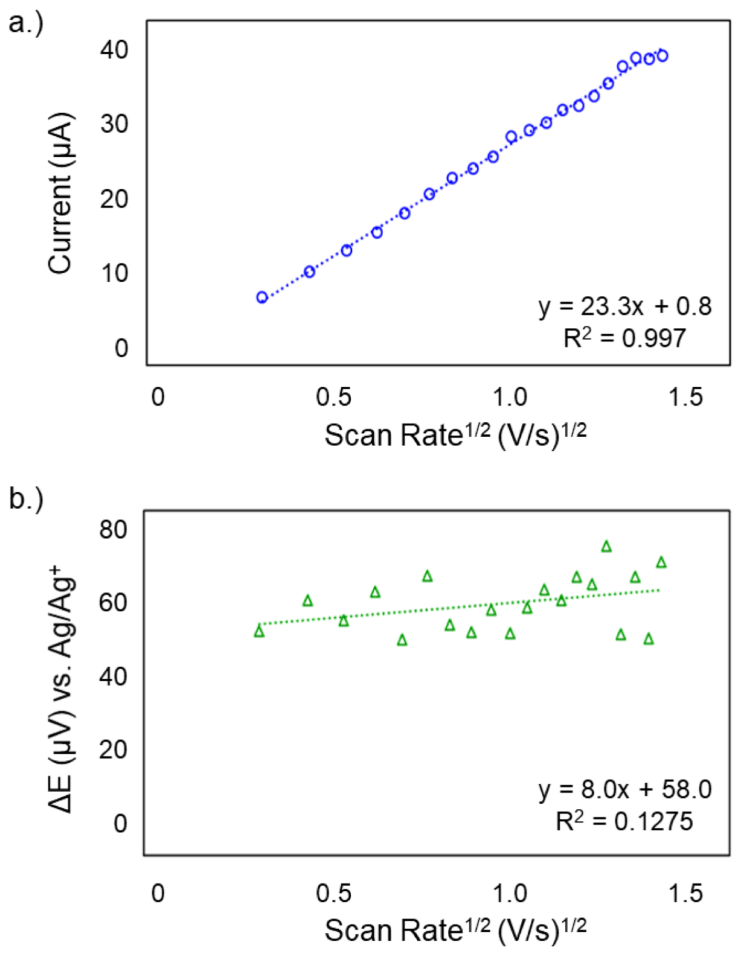

Figure S20. (a) Plots of i_p vs. $(v)^{1/2}$ and (b) ΔE vs. $(v)^{1/2}$ for the first oxidation event ($E_{1/2}$ = +0.22 V) of **6-$V_6O_7(OEt)_{12}$**

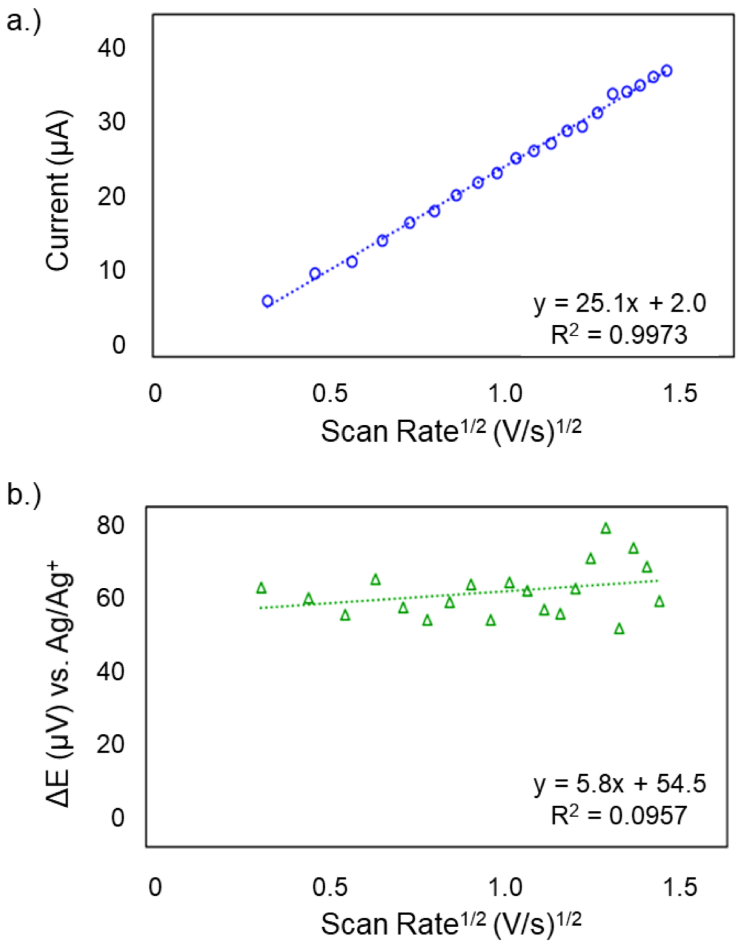

Figure S21. (a) Plots of i_p vs. $(v)^{1/2}$ and (b) ΔE vs. $(v)^{1/2}$ for the second oxidation event ($E_{1/2}$ = +0.79 V) of **6-$V_6O_7(OEt)_{12}$**

Figure S22. (a) absorbance spectra of **7-$V_6O_7(OEt)_{12}^{2-}$** at various concentrations in MeCN with 0.1 M $[NBu_4][PF_6]$ (b) Beer's Law plot of absorbance at 626 nm for **7-$V_6O_7(OEt)_{12}^{2-}$** in MeCN.

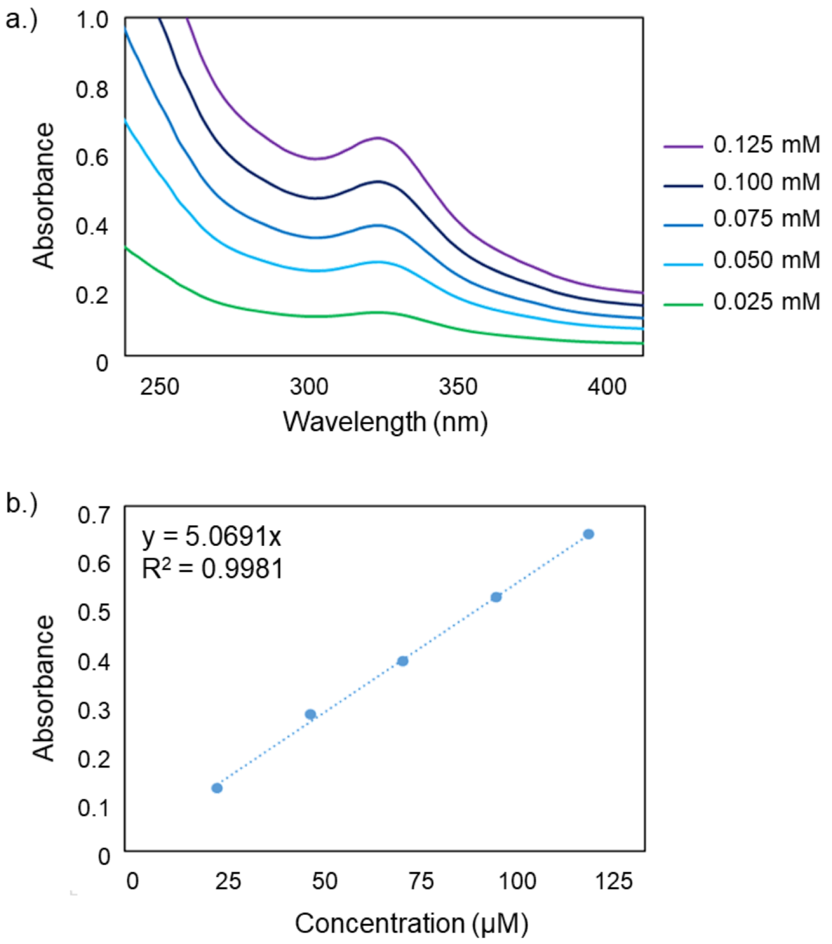

Molar Absorptivity at 323 nm: 5,069 $M^{-1}cm^{-1}$

Saturated Solution 1:
Diluted 2.5 uL to 10 mL MeCN
A = 0.13052
C = 2.575 E-5 M

10mL(2.575 E-5 M) = 2.5uL (X M)

X= 0.103 M

Saturated Solution 2:
Diluted 2.5 uL to 10 mL MeCN
A = 0.15207
C = 3.00 E-5 M

10mL(3.00 E-5 M) = 2.5uL (X M)

X= 0.120 M

Saturated Solution 3:
Diluted 2.5 uL to 10 mL MeCN
A =0.13813
C = 2.725 E-5 M

10mL(2.725 E-5 M) = 2.5 uL (X M)

X= 0.109 M

Average Solubility = 111 mM

Figure S23. (a) absorbance spectra of **8-$V_6O_7(OEt)_{12}^-$** at various concentrations in MeCN with 0.1 M $[NBu_4][PF_6]$ (b) Beer's Law plot of absorbance at 626 nm for **8-$V_6O_7(OEt)_{12}^-$** in MeCN.

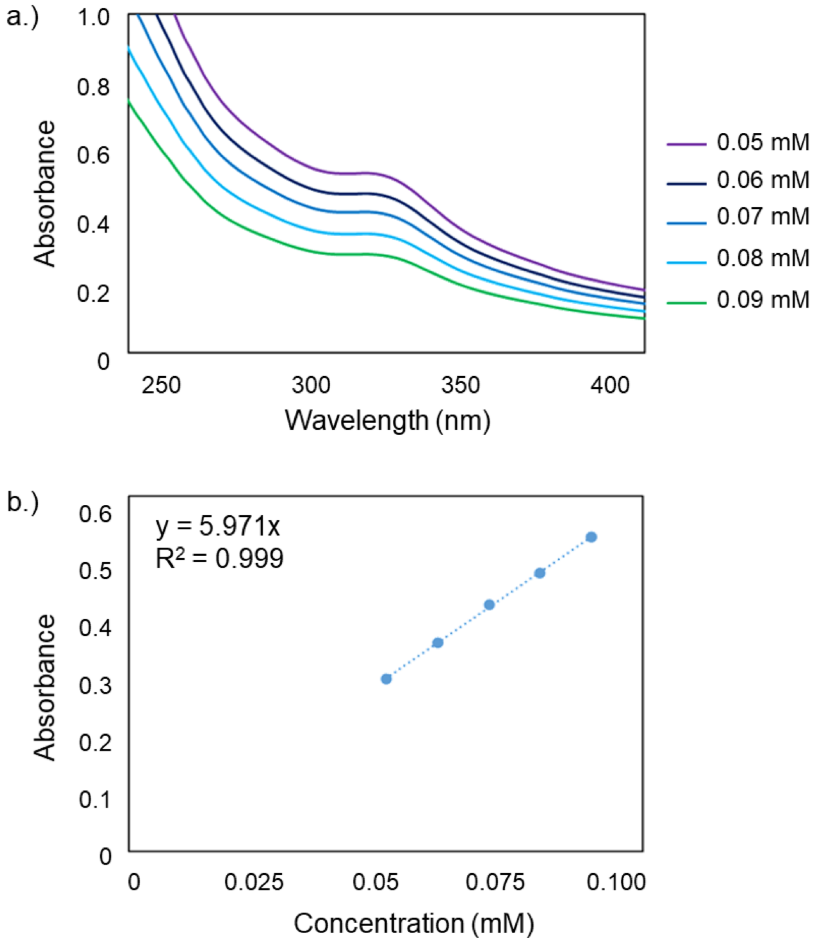

Molar Absorptivity at 325 nm: 5,971 $M^{-1}cm^{-1}$

Saturated Solution 1:
Diluted 2.5 uL to 10 mL MeCN
A = 0.34333
C = 5.750 E-5 M

10mL(5.750 E-5 M) = 2.5uL (X M)

X= 0.230 M

Saturated Solution 2:
Diluted 2.5 uL to 10 mL MeCN
A = 0.33587
C = 5.625 E-5 M

10mL(5.625 E-5 M) = 2.5uL (X M)

X= 0.225 M

Saturated Solution 3:
Diluted 2.5 uL to 10 mL MeCN
A =0.32691
C = 5.475 E-5 M

10mL(5.475 E-5 M) = 2.5 uL (X M)

X= 0.219 M

Average Solubility = 225 mM

Figure S24. (a) absorbance spectra of **6-$V_6O_7(OEt)_{12}$** at various concentrations in MeCN with 0.1 M $[NBu_4][PF_6]$ (b) Beer's Law plot of absorbance at 626 nm for **6-$V_6O_7(OEt)_{12}$** in MeCN.

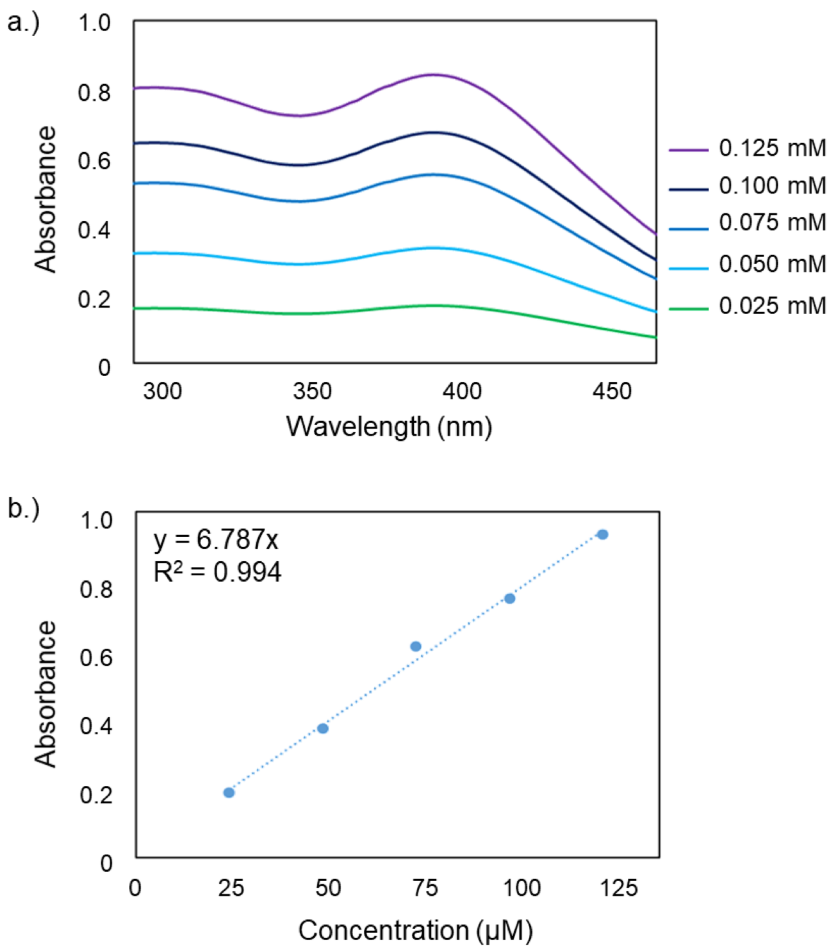

Molar Absorptivity at 325 nm: 6,787 $M^{-1}cm^{-1}$

Saturated Solution 1:
Diluted 10 uL to 10 mL MeCN
A = 0.29190
C = 4.301 E-5 M

10mL(4.301 E-5 M) = 10 uL (X M)

X= 0.043 M

Saturated Solution 2:
Diluted 10 uL to 10 mL MeCN
A = 0.37559
C = 5.534 E-5 M

10mL(5.534 E-5 M) = 10 uL (X M)

X= 0.055 M

Saturated Solution 3:
Diluted 10 uL to 10 mL MeCN
A =0.32224
C = 4.748 E-5 M

10mL(4.748 E-5 M) = 10 uL (X M)

X= 0.047 M

Average Solubility = 48 mM

Figure S25. (a) absorbance spectra of **9-$V_6O_7(OEt)_{12}^+$** at various concentrations in MeCN with 0.1 M $[NBu_4][PF_6]$ (b) Beer's Law plot of absorbance at 626 nm for **9-$V_6O_7(OEt)_{12}^+$** in MeCN.

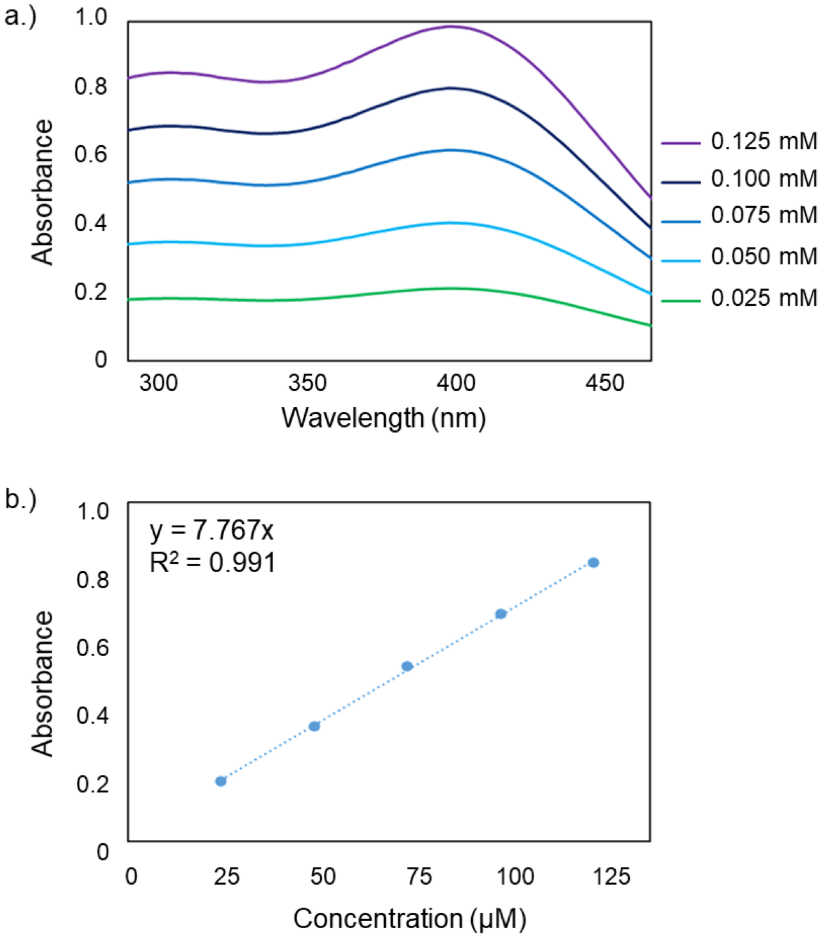

Molar Absorptivity at 325 nm: 7,767 $M^{-1}cm^{-1}$

Saturated Solution 1:
Diluted 2.5 uL to 10 mL MeCN
A = 0.38640
C = 4.975 E-5 M

10mL(4.975 E-5 M) = 2.5uL (X M)

X= 0.199 M

Saturated Solution 2:
Diluted 2.5 uL to 10 mL MeCN
A = 0.39619
C = 5.101 E-5 M

10mL(5.101 E-5 M) = 2.5uL (X M)

X= 0.204 M

Saturated Solution 3:
Diluted 2.5 uL to 10 mL MeCN
A =0.41553
C = 5.350 E-5 M

10mL(5.350 E-5 M) = 2.5 uL (X M)

X= 0.214 M

Average Solubility = 206 mM

Figure S26. (a) absorbance spectra of **10-$V_6O_7(OEt)_{12}^{2+}$** at various concentrations in MeCN with 0.1 M $[NBu_4][PF_6]$ (b) Beer's Law plot of absorbance at 626 nm for **10-$V_6O_7(OEt)_{12}^{2+}$** in MeCN.

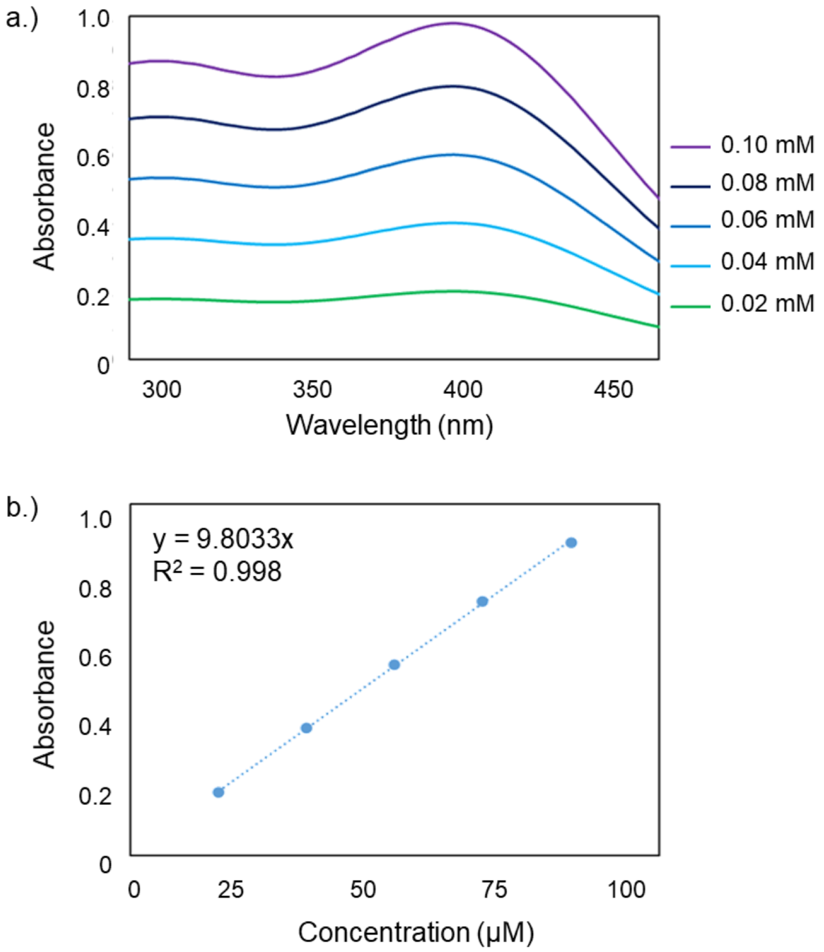

Molar Absorptivity at 325 nm: 9,803 $M^{-1}cm^{-1}$

Saturated Solution 1:
Diluted 2.5 uL to 10 mL MeCN
A = 0.31869
C = 3.251 E-5 M

10mL(3.251 E-5 M) = 2.5uL (X M)

X= 0.130 M

Saturated Solution 2:
Diluted 2.5 uL to 10 mL MeCN
A = 0.34555
C = 3.525 E-5 M

10mL(3.525 E-5 M) = 2.5uL (X M)

X= 0.141 M

Saturated Solution 3:
Diluted 2.5 uL to 10 mL MeCN
A =0.321048
C = 3.275 E-5 M

10mL(3.275 E-5 M) = 2.5 uL (X M)

X= 0.131 M

Average Solubility = 134 mM

Figure S27. (top) UV-vis in acetonitrile and (bottom) infrared absorption spectra of all charge states of **6-$V_6O_7(OEt)_{12}$**

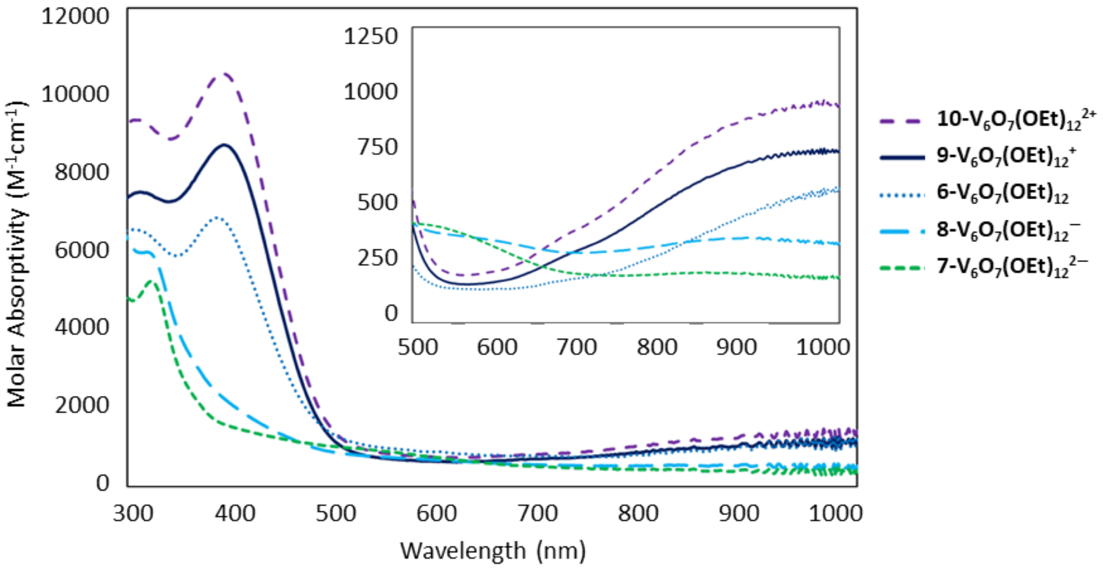

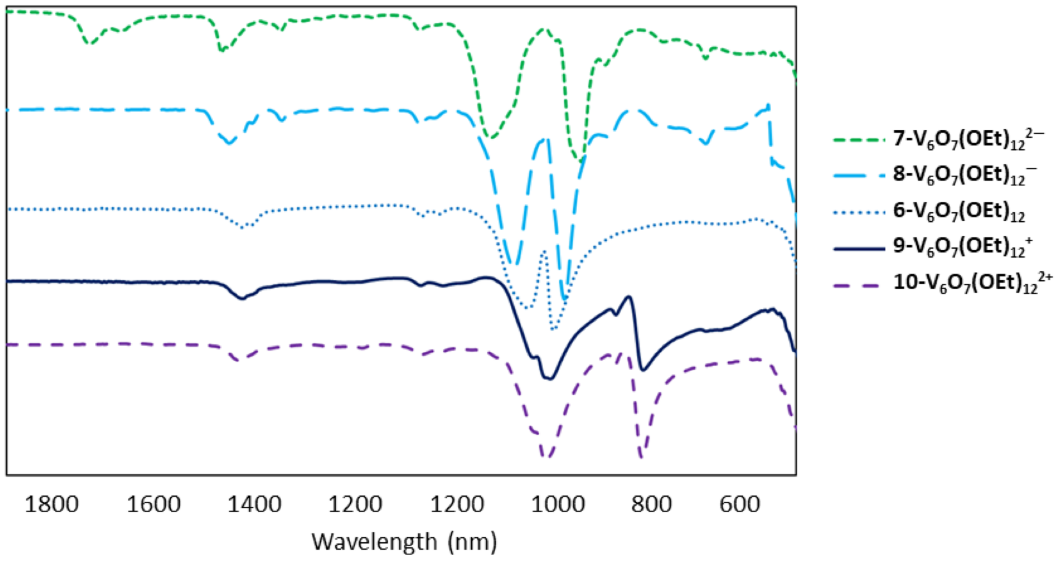

Figure S28. (a) Cyclic voltammograms at 100 mV/s and (b) absorption spectra of a 1 mM solution of **7-$V_6O_7(OEt)_{12}^{2-}$** with 0.1 M $[NBu_4][PF_6]$ in acetonitrile over the course of a week.

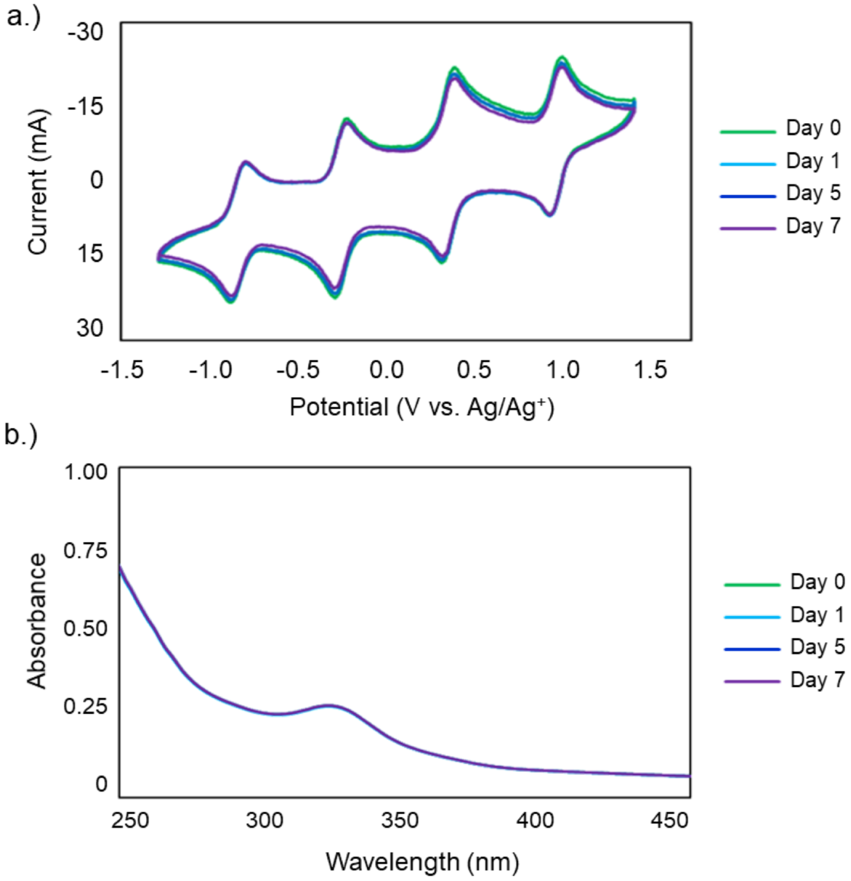

Figure S29. (a) Cyclic voltammograms at 100 mV/s and (b) absorption spectra of a 1 mM solution of **8-$V_6O_7(OEt)_{12}^-$** with 0.1 M $[NBu_4][PF_6]$ in acetonitrile over the course of a week.

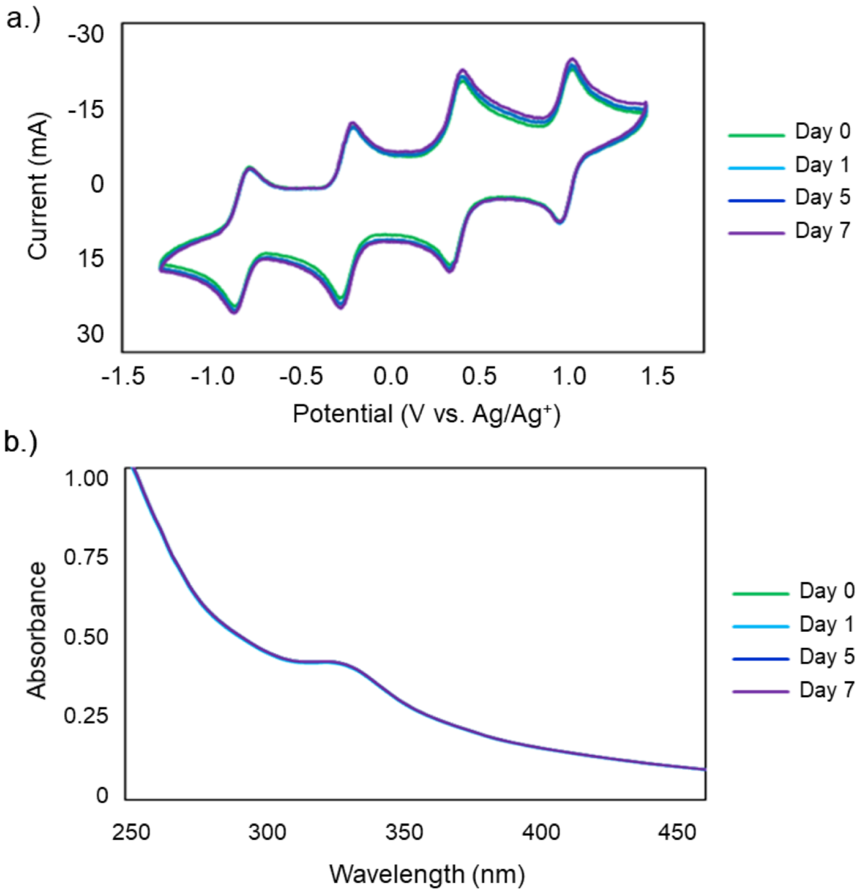

Figure S30. (a) Cyclic voltammograms at 100 mV/s and (b) absorption spectra of a 1 mM solution of **6-$V_6O_7(OEt)_{12}$** with 0.1 M $[NBu_4][PF_6]$ in acetonitrile over the course of a week.

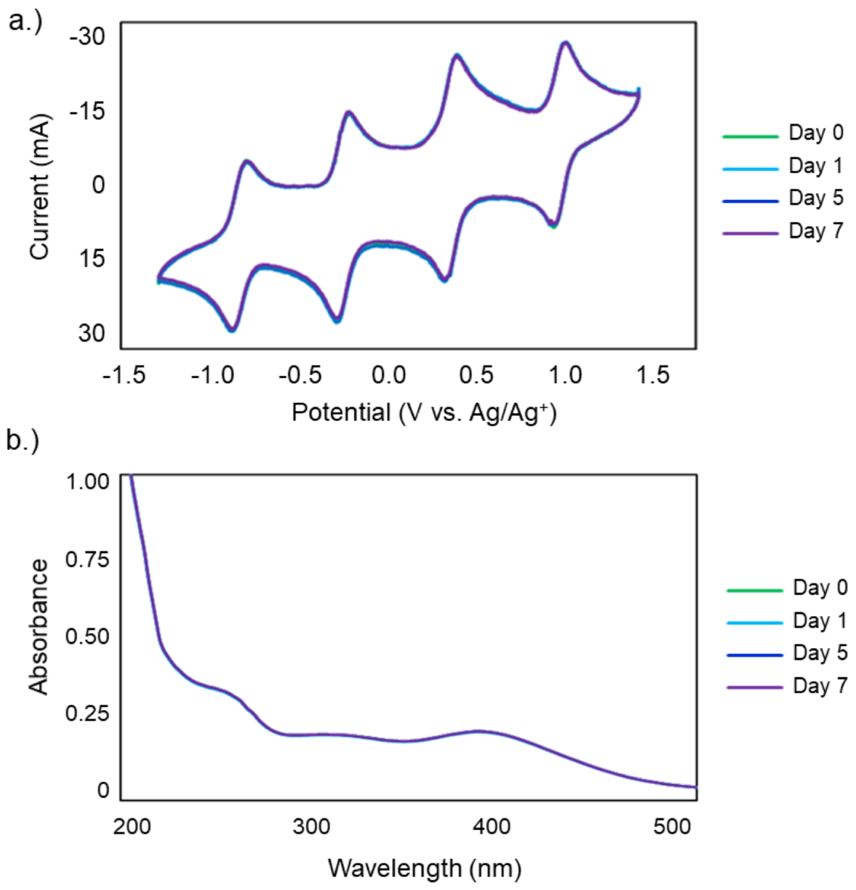

Figure S31. (a) Cyclic voltammograms at 100 mV/s and (b) absorption spectra of a 1 mM solution of **9-$V_6O_7(OEt)_{12}^+$** with 0.1 M $[NBu_4][PF_6]$ in acetonitrile over the course of a week.

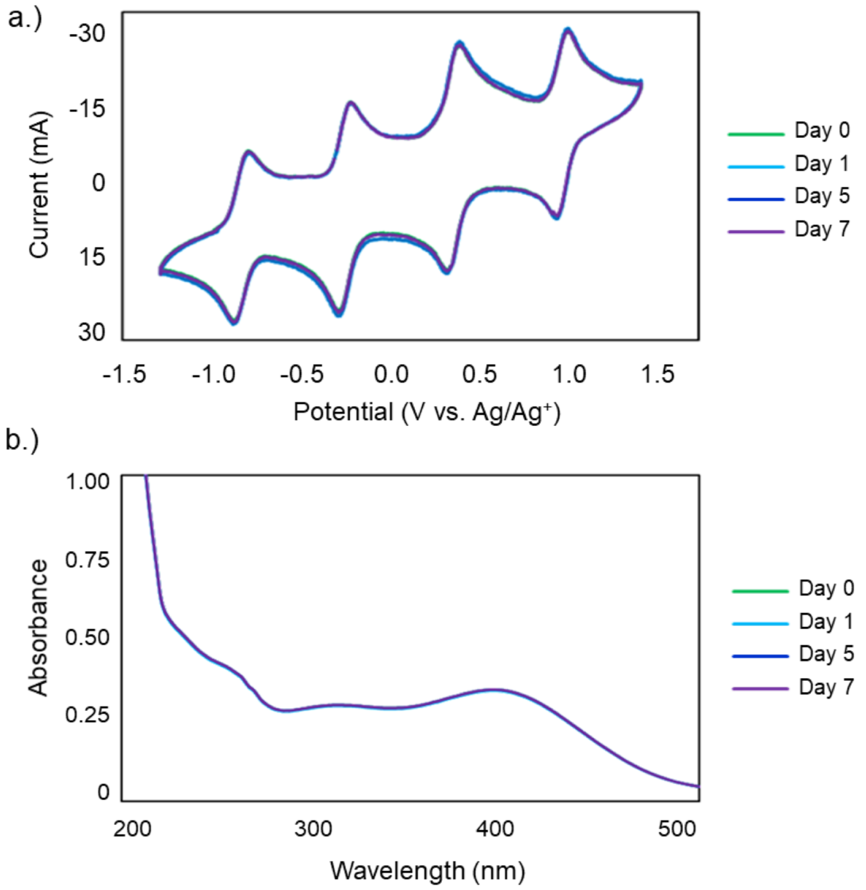

Figure S32. (a) Cyclic voltammograms at 100 mV/s and (b) absorption spectra of a 1 mM solution of **6-$V_6O_7(OEt)_{12}^{2+}$** with 0.1 M $[NBu_4][PF_6]$ in acetonitrile over the course of a week.

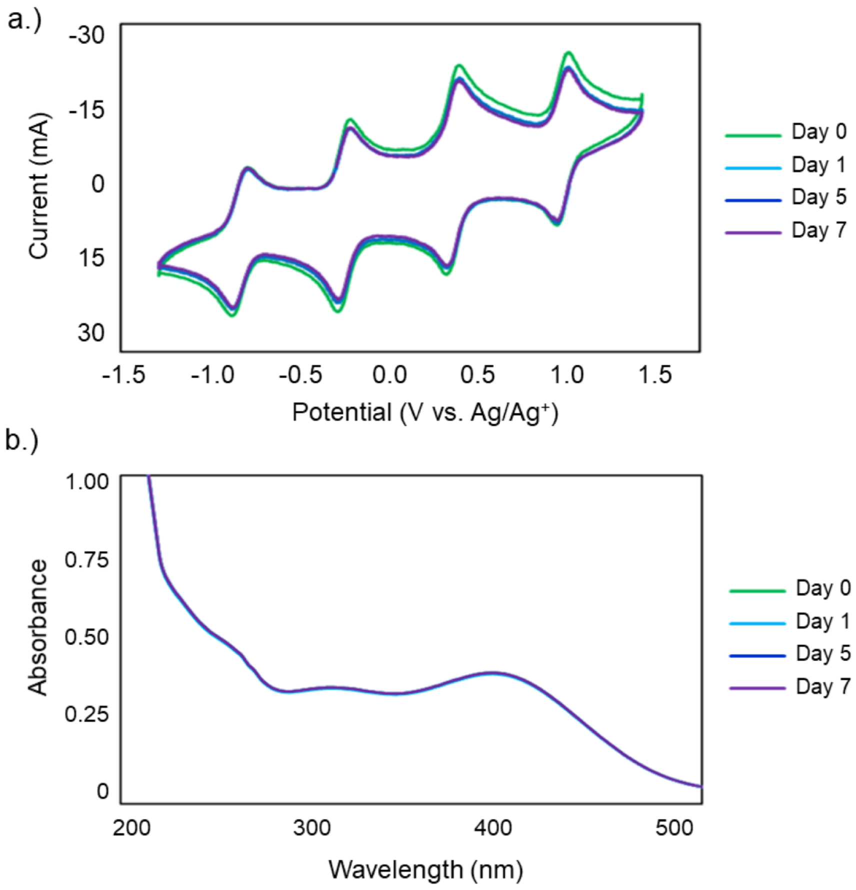

Figure S33. (top) Bulk oxidation (+1.1 V) of 0.1 mM **6-$V_6O_7(OEt)_{12}$** in acetonitrile with 0.1 M $[NBu_4][PF_6]$ supporting electrolyte and (bottom) CV of the solution before and after the oxidation

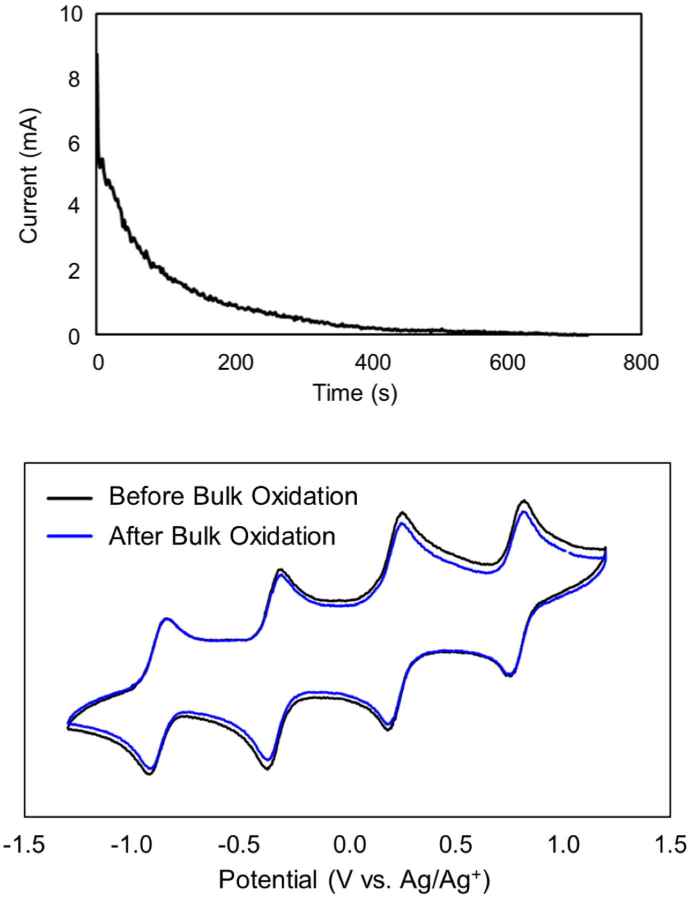

References:

1. Hoppe, E.; Limberg, C.; Ziemer, B., Mono- and Dinuclear Oxovanadium(V)calixarene Complexes and Their Activity as Oxidation Catalysts. *Inorganic Chemistry* **2006,** *45* (20), 8308-8317.
2. Daniel, C.; Hartl, H., Neutral and Cationic VIV/VV Mixed-Valence Alkoxo-polyoxovanadium Clusters [V6O7(OR)12]n+ (R = −CH3, −C2H5): Structural, Cyclovoltammetric and IR-Spectroscopic Investigations on Mixed Valency in a Hexanuclear Core. *Journal of the American Chemical Society* **2005,** *127* (40), 13978-13987.
3. Spandl, J.; Daniel, C.; Brüdgam, I.; Hartl, H., Synthesis and Structural Characterization of Redox-Active Dodecamethoxoheptaoxohexavanadium Clusters. *Angewandte Chemie International Edition* **2003,** *42* (10), 1163-1166.
4. (a) Liu, Q.; Sleightholme, A. E. S.; Shinkle, A. A.; Li, Y.; Thompson, L. T., Non-aqueous vanadium acetylacetonate electrolyte for redox flow batteries. *Electrochemistry Communications* **2009,** *11* (12), 2312-2315; (b) Sleightholme, A. E. S.; Shinkle, A. A.; Liu, Q.; Li, Y.; Monroe, C. W.; Thompson, L. T., Non-aqueous manganese acetylacetonate electrolyte for redox flow batteries. *Journal of Power Sources* **2011,** *196* (13), 5742-5745; (c) Kosswattaarachchi, A. M.; Friedman, A. E.; Cook, T. R., Characterization of a BODIPY Dye as an Active Species for Redox Flow Batteries. *ChemSusChem* **2016,** *9* (23), 3317-3323.
5. Nicholson, R. S.; Shain, I., Theory of Stationary Electrode Polarography. Single Scan and Cyclic Methods Applied to Reversible, Irreversible, and Kinetic Systems. *Analytical Chemistry* **1964,** *36* (4), 706-723.
6. Muhammad, H.; Tahiri, I. A.; Muhammad, M.; Masood, Z.; Versiani, M. A.; Khaliq, O.; Latif, M.; Hanif, M., A comprehensive heterogeneous electron transfer rate constant evaluation of dissolved oxygen in DMSO at glassy carbon electrode measured by different electrochemical methods. *Journal of Electroanalytical Chemistry* **2016,** *775*, 157-162.
